# Embryonic mesothelial-derived hepatic lineage of quiescent and heterogenous scar-orchestrating cells defined but suppressed by WT1

**DOI:** 10.1038/s41467-019-12701-9

**Published:** 2019-10-15

**Authors:** Timothy James Kendall, Catherine Mary Duff, Luke Boulter, David H. Wilson, Elisabeth Freyer, Stuart Aitken, Stuart John Forbes, John Peter Iredale, Nicholas Dixon Hastie

**Affiliations:** 1 MRC Human Genetics Unit, MRC Institute of Genetics & Molecular Medicine, The University of Edinburgh, Edinburgh, EH4 2XU UK; 2 University of Edinburgh Centre for Inflammation Research, The University of Edinburgh, Edinburgh, EH4 2XU UK; 3 MRC Centre for Regenerative Medicine, The University of Edinburgh, Edinburgh, EH4 2XU UK; 4 Senate House, University of Bristol, Bristol, BS8 1TH UK

**Keywords:** Mechanisms of disease, Cell lineage, Liver fibrosis

## Abstract

Activated hepatic stellate cells (aHSCs) orchestrate scarring during liver injury, with putative quiescent precursor mesodermal derivation. Here we use lineage-tracing from development, through adult homoeostasis, to fibrosis, to define morphologically and transcriptionally discreet subpopulations of aHSCs by expression of *WT1*, a transcription factor controlling morphological transitions in organogenesis and adult homoeostasis. Two distinct populations of aHSCs express *WT1* after injury, and both re-engage a transcriptional signature reflecting embryonic mesothelial origin of their discreet quiescent adult precursor. *WT1*-deletion enhances fibrogenesis after injury, through upregulated Wnt-signalling and modulation of genes central to matrix persistence in aHSCs, and augmentation of myofibroblastic transition. The mesothelial-derived lineage demonstrates punctuated phenotypic plasticity through bidirectional mesothelial-mesenchymal transitions. Our findings demonstrate functional heterogeneity of adult scar-orchestrating cells that can be whole-life traced back through specific quiescent adult precursors to differential origin in development, and define *WT1* as a paradoxical regulator of aHSCs induced by injury but suppressing scarring.

## Abstract (web summary)

Activated hepatic stellate cells of putative mesodermal origin orchestrate scarring during injury. Here, the authors define a discrete morphologically plastic lineage of embryonic mesothelial-derived scar-orchestrating cells, through a distinct quiescent adult precursor, defined and paradoxically inhibited by WT1.

## Introduction

Activated hepatic stellate cells (aHSCs) are the predominant scar-orchestrating myofibroblasts (MFBs) in fibrotic liver injury^1^ and have a critical role in primary liver carcinogenesis^2^. These mesenchymal cells derive from activation of perivascular quiescent HSCs (qHSCs)^3^. qHSCs are defined by retinoid storage but have diverse roles including vascular control^4^ and immunomodulation^5,6^. Activation of qHSCs involves retinoid remodelling, increased fibrogenic gene expression, and proliferation^7^.

Most fibrosis in adult chronic liver disease is within the deep parenchyma. A small proportion of subcapsular MFBs derive from the adult WT1-positive mesothelium after injury but the relevance of this is unclear^8^. A proportion of qHSCs in development originate from the mesoderm^9^ via hepatic mesothelium^10^. However, there remains uncertainty about the origin and heterogeneity of the majority of deep parenchymal aHSCs after adult injury and the functional significance of the developmental origin.

The hepatic mesothelium in both development and adulthood is characterised by Wilms’ Tumour 1 (*WT1*) expression^11^. *WT1* is a transcription factor with a critical role in organogenesis in both development^12^ and adult homoeostasis^13^. Critically, *WT1* regulates significant morphological plasticity, controlling mesenchymal states and, in many cases, defining mesenchymal origin^14,15^.

Given the relationship between mesenchyme and *WT1*, we examine *WT1* expression by HSCs in fibrotic liver disease. We define discreet populations of aHSCs whose origin we trace through adult precursors back to their ultimate embryological source. We show that mesothelial origin in development defines a specific population of qHSCs that is the precursor for distinct populations of aHSCs after adult injury. Further, *WT1* deletion permits morphological transition to a MFB phenotype and enhances fibrotic response in an in vivo model of pericentral fibrosis, defining *WT1* as a paradoxical negative regulator of fibrogenic capacity during injury.

## Results

### Embryonic origin defines two discrete populations of qHSCs

**Fig. 1:**
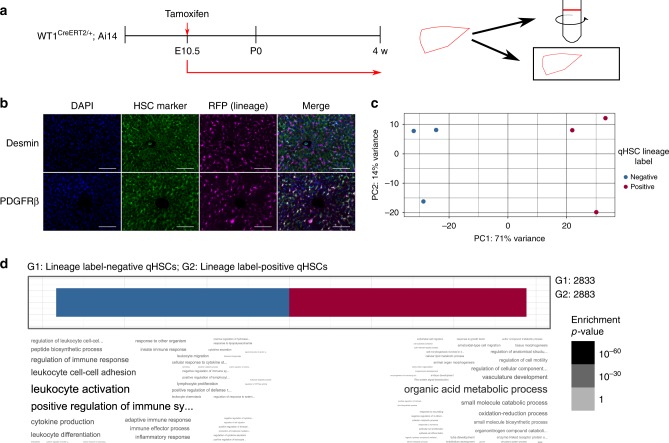
Functionally distinct subpopulations of qHSCs derive from alternative embryological origins. **a** Lineage labelling of hepatic mesothelial cells and their progeny was induced at E10.5 in WT1^CreERT2/+^;Ai14 animals, and qHSCs isolated or livers examined in adulthood. **b** Adult WT1^CreERT2/+^;Ai14 livers, labelled at E10.5, were stained for lineage label (RFP) and qHSC markers (desmin and PDGFRβ); 68.4 ± 3.46% qHSCs were lineage label positive (mean ± s.e.m., *n* = 3, scale bars 100 μm). **c** The presence of the lineage label was the major component describing variation in differentially expressed genes between lineage-labelled and unlabelled populations of qHSCs isolated from adult WT1^CreERT2/+^;Ai14 livers (*n* = 3), labelled at E10.5. **d** GO term analysis of differentially expressed genes between lineage-labelled and unlabelled qHSCs showed distinct functional profiles depending on origin

We sought to trace mesothelial-derived qHSCs from development through adult homoeostasis into injury to determine the full generational pathway of the most important scar-orchestrating cells. Developmental labelling was induced in WT1^CreERT2/+^;Ai14 mice at embryonic (E) day 10.5 (Fig. 1a), a time point restricting labelling in the liver to the mesothelium^10,16^. Labelled uninjured livers were stained for the lineage label (RFP) and an HSC marker (platelet-derived growth factor β (PDGFRβ), Fig. 1b). A subpopulation of 68.4 ± 3.46% of deep parenchymal qHSCs was lineage labelled (mean ± s.e.m, *n* = 3 animals). No RFP-positive cells were present in animals from control litters that had not received maternal tamoxifen administration.

qHSCs were isolated by density centrifugation from the livers of additional uninjured animals. Lineage label (RFP)-positive and -negative qHSCs were analysed by flow cytometry; 50.2 ± 3.8% were lineage-label positive (*n* = 11 animals). Representative samples were used for RNAseq (*n* = 3 animals). Principle component analysis demonstrated the separation of samples into two groups, with lineage label-status representing the dominant principal component (Fig. 1c). A total of 5716 genes were differentially expressed (Supplementary Data 1), corresponding to differential activation of 80 KEGG pathways (Supplementary Data 2).

In lineage label-positive qHSCs, gene ontology (GO) terms relating to morphogenesis and development were overrepresented (Fig. 1d), particularly those associated with the vasculature (‘vasculature development’, ‘endothelial cell migration’, ‘endothelium development’, and ‘circulatory system process’). In contrast, the lineage label-negative population was overrepresented by GO terms for responses to inflammation and immune regulation.

qHSCs are the major source of extracellular matrix (ECM) in the normal space of Disse, and expression of collagen and laminin by both lineage label-positive and -negative qHSCs was confirmed. However, the ECM components expressed by lineage label-positive and -negative cells were separate and complementary (Supplementary Table 1).

### Injury activates WT1-positive cells of mesothelial lineage

**Fig. 2:**
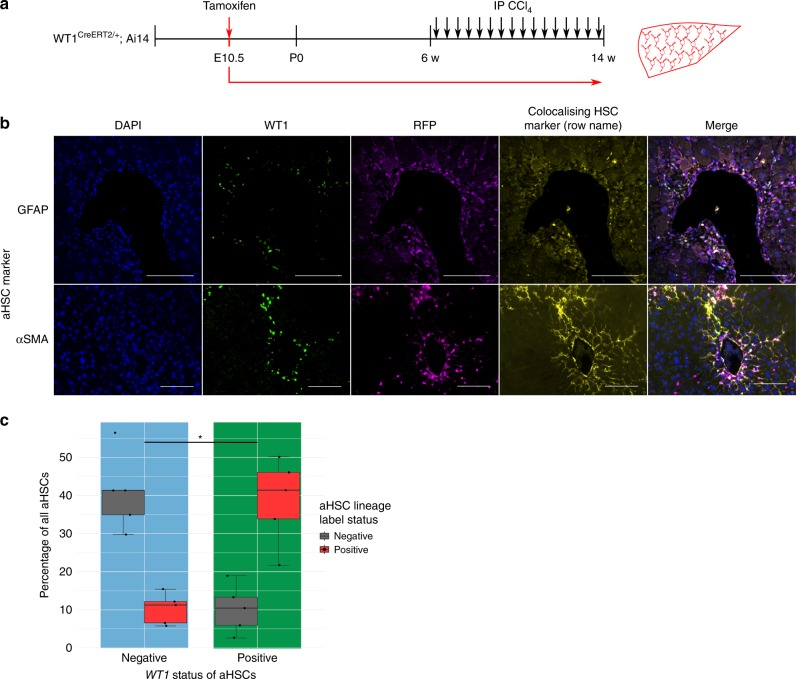
Adult injury induces a population of WT1-positive cells by activation of qHSCs that derive from the embryonic hepatic mesothelium. **a** Lineage labelling of hepatic mesothelium-derived cells was induced at E10.5 in a further cohort of WT1^CreERT2/+^;Ai14 animals and fibrosis induced by iterative injury with CCl_4_. **b** Lineage-labelled cells after injury were confirmed to be aHSCs by confocal microscopy demonstrating colocalization of the lineage label (RFP—lilac) with the HSC marker GFAP (yellow). Activated lineage-labelled cells were quantified after staining of injured livers for WT1 (green), lineage label (RFP—lilac), and αSMA (yellow) to demonstrate a population of WT1-positive activated cells originating from mesothelial-derived qHSCs. **c** 78.1 ± 5.3% of WT1-positive cells were lineage labelled, compared with 20.5 ± 3.7% of WT1-negative cells (Welch two-sample *t*-test *t*(7.144) = −7.9601, *p = 8.437 × 10^–5^, *n* = 5 animals, data are represented as individual points with median (centre line), first and third quartiles (lower and upper box limits), 1.5× interquartile range (whiskers). Scale bars 100 μm

Adult male WT1^CreERT2/+^;Ai14 offspring, labelling induced at E10.5, were chronically injured with carbon tetrachloride (CCl_4_) injection (Fig. 2a). Livers were stained for the lineage label (RFP) and α-smooth muscle actin (αSMA); a subpopulation representing 53.7 ± 10.4% of aHSCs carried the lineage label (*n* = 5 animals).

Given this indicated origin by activation of qHSCs derived from the WT1-positive mesothelium, and the association of WT1 with morphological plasticity, livers were further stained for WT1 alongside the lineage label (RFP) and either another HSC marker (glial fibrillary acidic protein (GFAP)) or αSMA (Fig. 2b). In uninjured control liver, there were no WT1-positive cells within lobules (Supplementary Fig. 1); only the hepatic mesothelium and rare cells located immediately beneath were WT1-positive. After injury, the lineage-label persisted in colocalizing with the HSC marker, but pericentral cells in the areas of scarring were WT1-positive; 78.1 ± 5.3% of WT1-positive αSMA-positive cells carried the lineage label compared with 20.5 ± 3.7% of WT1-negative αSMA-positive cells (Fig. 2c; Welch two-sample *t*-test *t*(7.144) = −7.9601, *p* = 8.437 × 10^–5^, 10 fields/animal, *n* = 5 animals). Given the efficiency of both Cre-labelling and identification of labelled cells by immunofluorescence, this indicates extensive overlap between cells from a mesothelial-derived lineage and WT1-positive cells after injury, and their membership of a common lineage.

### *WT1* expression defines an aHSC subpopulation in fibrosis

**Fig. 3:**
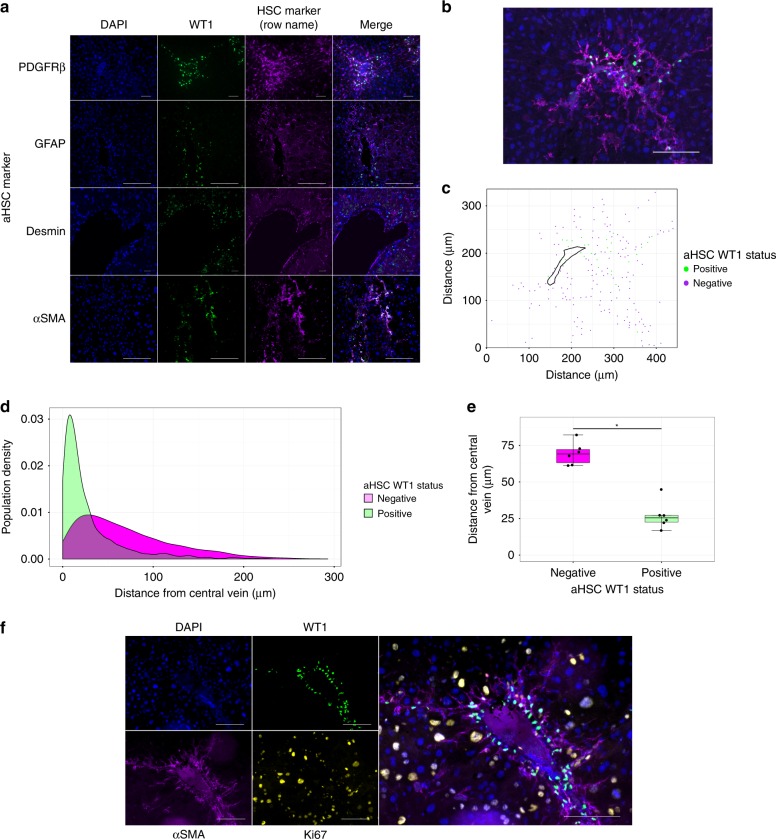
Subpopulations of aHSCs defined by WT1 expression are distributed differentially within hepatic scars and show differences in cell cycling. **a** WT1-positive cells are present in murine liver injured by CCl_4_ injection, colocalizing with markers of aHSCs (rows show colocalized staining with PDGFRβ, GFAP, desmin, and αSMA). Scale bars 100 μm. **b** The nuclear position of WT1-positive and WT1-negative aHSCs, and hepatic vein profile were marked in each image of liver from animals chronically injured with CCl_4_ to allow quantification of the fibrospatial distribution of aHSCs (**c**). Scale bars 100 μm. **d** The distances from nuclei to central vein were calculated for each biological replicate, demonstrating different distributions within hepatic scars of WT1-positive and WT1-negative aHSCs (representative density distribution plot, bootstrapped Kolmogorov–Smirnov test *p* < 0.00001). **e** The mean distances of WT1-positive and WT1-negative aHSCs to the central vein, on a per animal basis, were calculated to show that WT1-positive cells were significantly closer to central vein profiles (*, Welch two-sample *t*-test, *t*(9.6195) = 8.4046, *p* = 0.0000098, *n* = 6). Data are represented as individual points with median (centre line), first and third quartiles (lower and upper box limits), 1.5× interquartile range (whiskers). **f** Livers injured with chronic CCl_4_ injection were stained for WT1, Ki67, and αSMA. Ki67 immunopositivity was significantly greater for WT1-negative compared with WT1-positive aHSCs (Welch two-sample *t*-test, *p* < 0.001, 20.3 ± 0.9% versus 1.9 ± 0.7%, 10 pericentral fields/animal, *n* = 3). Scale bars 100 μm

To characterise the WT1-positive αSMA-positive cells in injured liver further, fibrotic tissue from a range of injury models was examined. Large numbers of WT1-positive cells were present in fibrotic liver in all models of injury. WT1-positive cells were present in models with pericentral injury (CCl_4_, Fig. 3a), mixed portal and lobular injury (thioacetamide (TAA)), and biliary injury (bile duct ligation (BDL) and 3,5-diethoxycarbonyl-1,4-dihydrocollidine (DDC) diet, Supplementary Fig. 1).

WT1-positive cells were present within areas of fibrosis corresponding to the model-specific pattern of injury. For example, after chronic injury with CCl_4_, WT1-positive cells were located pericentrally, but no WT1-positive cells were present within or adjacent to the uninjured portal tracts (Supplementary Fig. 1).

WT1 immunopositivity was restricted to cells expressing αSMA, GFAP, desmin, and PDGFRβ), identifying them as aHSCs (Fig. 3a). There was no expression in cells expressing cytokeratins, or with hepatocellular or biliary epithelial morphology (Supplementary Fig. 1).

Human liver with a spectrum of fibrotic diseases was examined (Supplementary Fig. 1). Within the parenchyma, *WT1* expression was restricted to αSMA-positive cells within hepatic scars in all cases.

### aHSC populations vary in distribution and cell-cycle state

We sought to determine the topographical distribution of subpopulations of aHSCs, defined by WT1-status, within scars. Sections of liver from wild-type animals chronically injured with CCl_4_ were stained for WT1 and αSMA; 45.6 ± 2.3% of pericentral αSMA-positive aHSCs were WT1 positive; 92.6 ± 1.6% of WT1-positive cells were αSMA positive (1405 αSMA-positive cells counted, 10 fields total, *n* = 3 animals). Only rare pericentral WT1-positive cells were αSMA negative.

The nuclear positions of WT1-positive and WT1-negative aHSCs were marked, and the central vein circumference defined (10 pericentral fields/animal, *n* = 6 animals). The distance of each αSMA-positive cell nucleus to the closest point on the central vein circumference was calculated (Fig. 3b and c). The WT1-positive subpopulation of aHSCs was present throughout scars but a significantly larger proportion was closer to central veins compared with more evenly distributed WT1-negative aHSCs (Fig. 3d, bootstrapped Kolmogorov–Smirnov test for each animal, *p* < 0.00001). The mean distance of WT1-positive aHSCs to central vein was 27.0 ± 9.6 μm compared with 69.4 ± 7.8 μm for WT1-negative cells (Fig. 3e, Welch two-sample *t*-test, *t*(9.6195) = 8.4046, *p* = 0.0000098).

Sections of liver from wild-type animals chronically injured with CCl_4_ were also stained for Ki67 alongside WT1 and αSMA. The proportion of aHSCs immunopositive for the proliferation marker Ki67 was significantly greater for WT1-negative compared with WT1-positive aHSCs (Fig. 3f, Welch two-sample *t*-test *p* < 0.001, 20.3 ± 0.9% versus 1.9 ± 0.7%, 10 pericentral fields/animal, *n* = 3 animals).

### Acute injury rapidly induces WT1-positive aHSC populations

De novo activation of qHSCs was induced in wild-type animals by a single CCl_4_ injection (0.25 μl g^−1^ body weight). Rare WT1-positive aHSCs were present 3 days after injury (0.33 ± 0.33 WT1-positive cells/10 central vein profiles/animal) and in larger numbers after 7 days (104.67 ± 4.67 WT1-positive cells/10 central vein profiles/animal, *n* = 3 animals).

A larger dose of CCl_4_, causing greater hepatocellular necrosis (Supplementary Fig. 2), induced more abundant WT1-positive cells 3 days after injury. There was a strong positive correlation between administered dose and WT1-positive cell number (Spearman’s rank correlation *r*_s_ = 0.8911, *p* = 0.01713, *n* = 3 animals, Supplementary Fig. 2).

### WT1 defines morphologically distinct aHSC subpopulations

To allow the morphological properties of aHSC subpopulations to be examined, an in vitro model of activation by culture on plastic was used. A green fluorescent protein (GFP) reporter line (WT1^GFP/+^) allowed isolation of WT1-positive cells^17^. qHSCs from livers of uninjured WT1^GFP/+^ mice were isolated by field-standard density centrifugation based upon buoyancy that reflects the lipid content specific to HSCs. Cells derived by this method are accepted as meriting designation as HSCs. Isolated cells were cultured on plastic for 7 days to full activation.

**Fig. 4:**
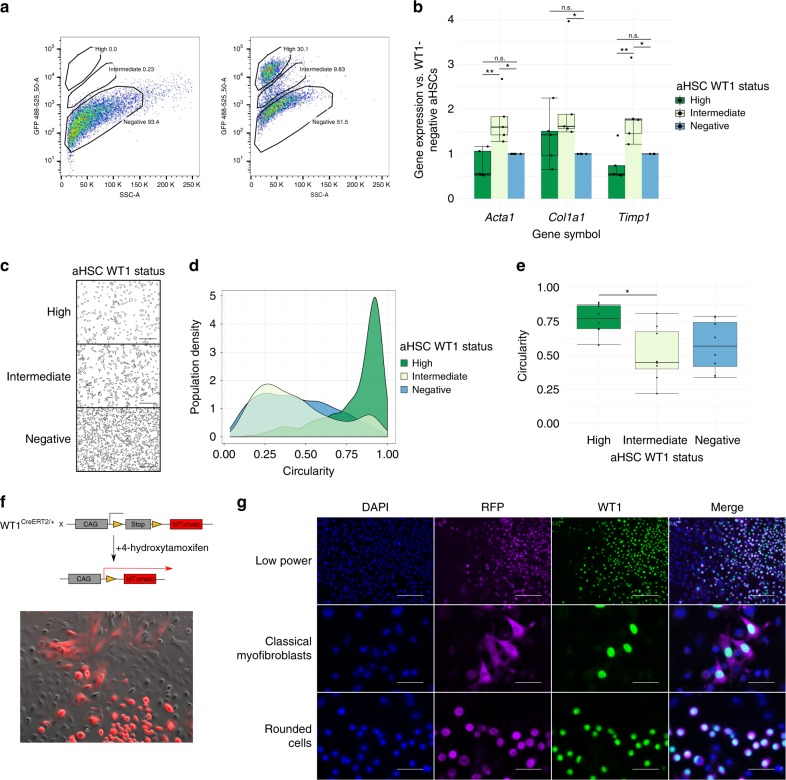
WT1 expression defines morphologically distinct subpopulations of aHSCs. **a** Quiescent HSCs from WT1^GFP/+^ animals were activated by 7 days of culture on plastic and examined by flow cytometry, demonstrating three distinct subpopulations based on GFP expression (wild-type cells left panel, WT1^GFP/+^ cells right panel, representative plots with population percentages). **b** All three subpopulations were profibrogenic but the WT1-intermediate population demonstrated enhanced fibrogenic gene expression (one-way ANOVA *(Acta1* *F*(2,12) = 11.72, *p* = 0.0015; *Timp1* *F*(2,12) = 11, *p* = 0.0019; *Col1a1* *F*(2,12) = 4.496, *p* = 0.0349; post-hoc Tukey, *< 0.05, ** < 0.01, *n* = 5, *n*,s *p* > 0.05). Data are represented as individual points with median (centre line), first and third quartiles (lower and upper box limits), 1.5× interquartile range (whiskers). **c** WT1-high aHSCs are round in profile, whilst WT1-intermediate and WT1-negative cells are myofibroblast-shaped. Scale bars 100 μm. **d** Representative population distribution of ‘circularity’ from a single cell preparation. **e** The circularity of subpopulations of aHSCs based on WT1 status was significantly different; post-hoc Tukey testing indicated that the circularity of WT1-high cells was significantly different to that of WT1-intermediate cells (*, one-way ANOVA *F*(2,21) = 4.997, *p* = 0.0168, *n* = 8). Data are represented as individual points with median (centre line), first and third quartiles (lower and upper box limits), 1.5× interquartile range (whiskers). **f** Morphologically biphasic WT1-positive cells in unsorted aHSC cultures of 4-hydroxytamoxifen-treated WT1^CreERT2/+^;Ai14 cells, subsequently fixed and stained for RFP and WT1 (**g**, scale bars 200 μm (upper row), 50 μm (middle and lower rows), representative of three biological replicates)

By flow cytometry, <0.1% of viable cells present immediately after isolation but before activation by culture on plastic were GFP positive (Supplementary Fig. 3). In fully activated cultures, 30.4 ± 4.6% of cells were WT1 positive (*n* = 12 animals). Within the WT1-positive population, two distinct populations were identified; 49.3 ± 6.7% of WT1-positive cells were GFP-high and the remainder GFP-intermediate (Fig. 4a).

*WT1* expression of sorted subpopulations by qPCR correlated with GFP intensity (Spearman’s rank correlation *r*_s_ = 0.962, *p* = 1.009 × 10^–8^, *n* = 5 animals, Supplementary Fig. 3). All subpopulations expressed the well-characterised fibrogenic activation markers *Acta1*, *Timp1*, and *Col1a1* (Fig. 4b). For each gene, there was a statistically significant difference between groups determined by one-way ANOVA (*Acta1* *F*(2,12) = 11.72, *p* = 0.0015; *Timp1* *F*(2,12) = 11, *p* = 0.0019; *Col1a1* *F*(2,12) = 4.496, *p* = 0.0349). The WT1-intermediate subpopulation showed higher expression by post-hoc Tukey testing (*p* < 0.05).

The number of mesothelial cells from the surface monolayer is low compared to the diffuse parenchymal population of qHSCs, making it highly unlikely that mesothelial cells could significantly contaminate a selective HSC isolation. Further, there is no cogent biological reason for mesothelial cell buoyancy to permit contamination of the HSC isolate. However, we sought to exclude the possibility of mesothelial contamination by labelling the surface of the liver prior to liver digestion. qHSCs were isolated from WT1^GFP/+^ animals after permanent fluorescent labelling of surface mesothelial cells in vivo (*n* = 3 animals, Supplementary Fig. 3). Labelled contaminating cells were not found in cultures of isolated HSCs during activation.

WT1-defined subpopulations of aHSCs from further WT1^GFP/+^ animals, generated by culture on plastic, were obtained by FACS. The WT1-high, WT1-intermediate, and WT1-negative subpopulations were plated separately back onto plastic and allowed to adhere overnight.

WT1-high cells were round, whilst WT1-intermediate and WT1-negative cells were classical MFB-shaped (Fig. 4c). Morphometric analysis confirmed that the population distribution of WT1-high aHSC ‘circularity’ was distinct from that of both WT1-intermediate and WT1-negative aHSCs (Fig. 4d, *p* < 0.0001, boot-strapped Kolmogorov–Smirnov test for each replicate). Using multiple biological replicates, a statistically significant difference in mean cell circularity between groups was confirmed (Fig. 4e, one-way ANOVA *F*(2,21) = 4.997 *p* = 0.0168, *n* = 8 animals).

To exclude the possibility that morphological differences were a biophysical artefact of isolation by FACS, the morphology of unsorted populations was examined using a WT1^CreERT2/+^;Ai14 reporter line^18,19^ (Fig. 4f). RFP expression in aHSCs was evident in half the cultured cells 24 h after Cre induction at 6 days of culture on plastic. Two morphologies of labelled cells were evident, rounded and classical MFB-shaped. In contrast, most WT1(RFP)-negative cells were classical MFB-shaped. Similarly treated cells were fixed and stained for WT1 and RFP to confirm efficient label induction in WT1-positive cells and demonstrate the two distinct populations of WT1-positive aHSCs (Fig. 4g).

### *WT1* status defines discrete aHSC transcriptional profiles

**Fig. 5:**
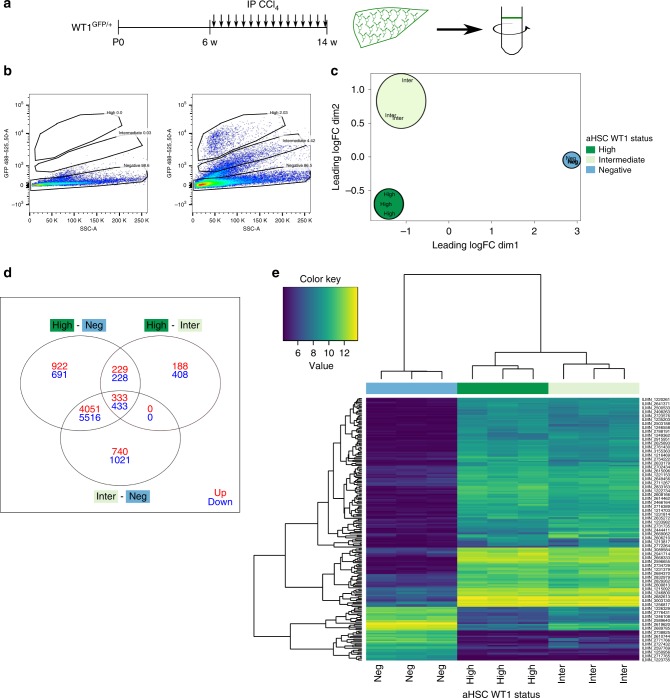
Subpopulations of aHSCs defined by WT1 expression in chronic fibrotic injury in vivo are transcriptionally distinct. **a** WT1^GFP/+^ animals were iteratively injured with CCl_4_ to induce HSC activation and fibrosis prior to isolation of subpopulations defined by WT1(GFP) expression by flow cytometry (*n* = 3 animals). **b** Subpopulations of WT1(GFP)-high and WT1(GFP)-intermediate were present within the isolated HSC fraction from injured WT1^GFP/+^ animals (right panel) but not injured wild-type animals (left, representative plots with population percentages). **c** A multi-dimensional scaling plot of gene expression after microarray analysis of WT1-positive in vivo activated and WT1-negative in vitro aHSCs demonstrated separation based on WT1(GFP) expression. **d** Over and underexpressed probes between subpopulations of aHSCs determined by fitting of a linear model. **e** Hierarchical clustering independently separated subpopulations into groups defined by WT1 expression

To examine subpopulations of aHSCs in vivo, a cohort of adult male WT1^GFP/+^ mice were chronically injured with CCl_4_ (Fig. 5a). The two distinct populations of WT1-positive cells present after activation in vitro were confirmed in vivo by flow cytometry of freshly isolated HSCs from fibrotic liver (Fig. 5b); the WT1-positive subpopulations represented a much smaller proportion of all isolated HSCs, as expected, given the presence in the buoyant HSC fraction of the large majority of qHSCs from uninjured parenchyma in addition to the WT1-negative aHSCs. The transcriptomes of in vivo injury-generated WT1-positive subpopulations was examined by microarray (*n* = 3 animals).

A multi-dimensional scaling plot of raw expression data demonstrated clustering of aHSC populations based on *WT1* status (Fig. 5c). Hierarchical clustering independently separated populations into groups defined by *WT1* expression, with the WT1-high and WT1-intermediate subpopulations of aHSCs more closely related to one another than either was to the WT1-negative population (Fig. 5d, e, Supplementary Tables 2–7, Supplementary Data 3–5).

The top overexpressed genes in both WT1-positive populations included part of a mesothelial gene signature (*Dmkn*, *Upk1b*, *Upk3b*, *Msln*, *Gpm6a*)^20^.

**Fig. 6:**
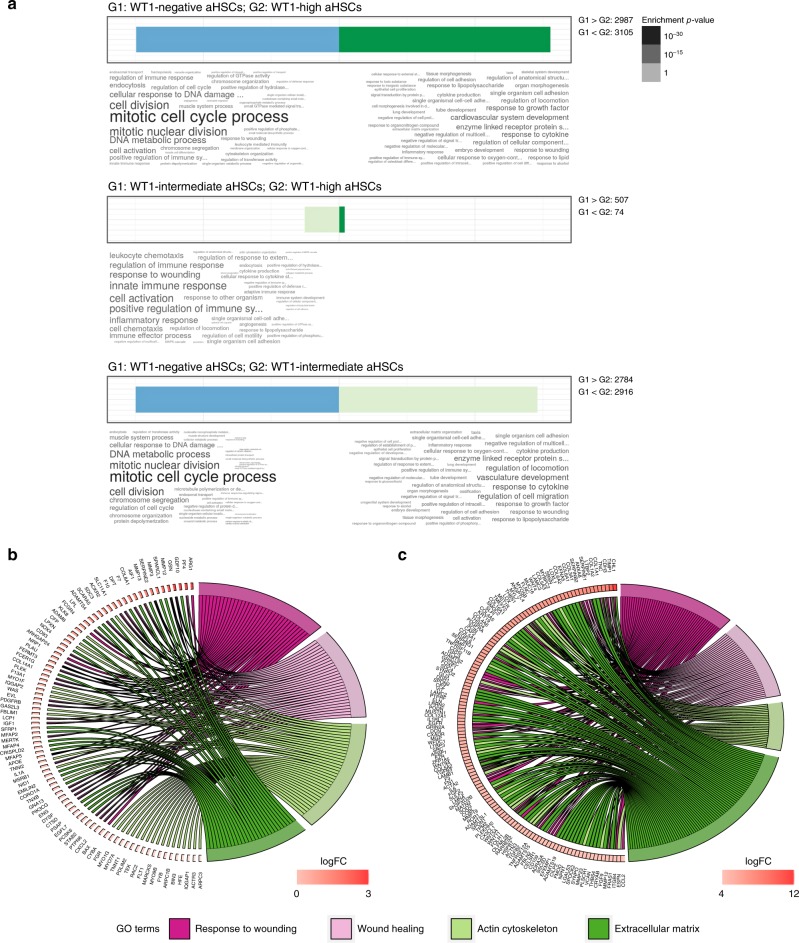
The transcriptional profiles of aHSC populations defined by WT1 expression are classically activated but distinct, with enhanced scar-related processes in WT1-intermediate cells. **a** GO term analysis of differentially expressed genes between subpopulations of aHSCs defined by WT1 expression demonstrate significant differences in profiles between all subpopulations, with WT1-high and WT1-intermediate populations showing fewer differences. **b** Specific mapping of differentially expressed genes to GO terms for indicative scarring responses demonstrate the enhancement in WT1-intermediate aHSCs compared with WT1-high aHSCs. **c** The transcriptome, determined by RNAseq, of aHSCs isolated from PDGFRβCre;WT1^GFP/+^ animals (*n* = 6) with liver fibrosis induced by iterative injury with CCl_4_ was compared with that of quiescent lineage-label positive HSCs from WT1^CreERT2/+^;Ai14 animals (*n* = 3) induced at E10.5. Gene ontology terms mapped to differentially expressed genes for WT1-high cells demonstrate engagement of cellular processes associated with the HSC activation paradigm

GO term differences between the morphologically distinct WT1-high and WT1-intermediate aHSCs were accounted for almost exclusively by overrepresentation in WT1-intermediate cells of terms relating to immune response and regulation, cell activation, response to wounding and inflammation, and chemotaxis (Fig. 6a). This corresponded with activation of KEGG pathways representing responses to inflammation, infection, and injury (Supplementary Table 5) in WT1-intermediate cells. Explicit mappings of differentially expressed genes to scarring-related GO terms, showing upregulation in WT1-intermediate cells in keeping with greater fibrogenic capacity, are shown in Fig. 6b, including matrix metalloproteinases, chemokine ligands, chemokine receptors, and other genes implicated in fibrogenesis; *Col1a1*, *Col1a2*, *Timp1*, and *Acta2* are not differentially expressed. Of note, a number of the genes whose expression is upregulated in the WT1-intermediate aHSCs have been shown to be central to tissue scarring in mechanistic studies, including *Pdgfrb*^21^, *Tgfb*^22^, *Pf4* (Cxcl4)^23^, *Ccl6*^24^, *Ccl11*^25^, *Mmp12*^26^, and *Mmp3*^27,28^ (Supplementary Table 8). This suggests that the transcriptionally distinct populations of aHSCs generated by in vivo injury may influence fibrogenesis by mechanisms more complex than simple regulation of the transcription of genes encoding matrix components.

There was common overrepresentation in both WT1-positive populations of terms relating to cell activation, tissue morphology, ECM, and cell adhesion. This corresponded to activation of the KEGG pathway ‘ECM-receptor interaction’ and relative inactivation of ‘NF-kappa B signalling’ in both WT1-positive populations compared with WT1-negative cells (Supplementary Tables 6 and 7). Terms relating to cell cycling and proliferation were overrepresented in the WT1-negative population, as expected given the significantly higher Ki67 immunopositivity of WT1-negative aHSCs.

Colocalization of cytoplasmic GFP and nuclear WT1 was confirmed in aHSCs and the mesothelium (Supplementary Fig. 4). Colocalization in podocytes (in which *WT1* is highly expressed) was also confirmed. As expected, GFP-positive cells in fibrotic livers also expressed αSMA.

To validate the microarray data, qPCR for 12 genes covering the full range of differential expression (WT1-high versus WT1-negative) was undertaken. There was a strong positive correlation between differential gene expression determined by each method (*r*_s_ = 0.945, *p* = 2.443 × 10^–6^, Supplementary Fig. 4). The same validation set was assessed in three paired populations of WT1-high and WT1-negative aHSCs isolated by flow cytometry after in vitro activation of qHSCs from WT1^GFP/+^ animals. There was a strong positive correlation of differential gene expression between populations of aHSCs generated by in vivo injury and in vitro activation (*r*_s_ = 0.839, *p* = 0.001192), confirming the validity of in vitro generation of subpopulations of aHSCs as a model for activation of qHSC in vivo (Supplementary Fig. 4).

### Embryonic mesothelial lineage aHSCs are classically active

Transcriptomic data from mesothelial-lineage label positive qHSCs and WT1-positive aHSCs deriving from them after in vivo injury were interrogated to define the transcriptional changes of this discrete lineage. A total of 7406 genes were differentially expressed between lineage-positive qHSCs and WT1-high aHSCs, including 134 genes upregulated and 291 genes downregulated in WT1-high aHSCs by at least 100-fold. A total of 5884 genes were differentially expressed between lineage-positive qHSCs and WT1-intermediate aHSCs, including 70 genes upregulated and 117 genes downregulated in WT1-high aHSCs by at least 100-fold.

Both WT1-positive subpopulations of aHSCs show differential upregulation of genes mapped to GO terms accepted as part of the existing HSC activation paradigm, including ‘wound healing’, ‘extracellular matrix’, ‘actin cytoskeleton’, ‘inflammatory response’, and ‘cytokine production’, supporting their designation as a defined lineage within this category of scar-orchestrating cells (Supplementary Fig. 5). Individual genes mapping to these key terms are illustrated in Fig. 6c and Supplementary Fig. 5 (complete list in Supplementary Data 6 and 7). There is significant downregulation of Ctrb1 (chymotrypsinogen B1) in both WT1-high and WT1-intermediate aHSCs compared with qHSCs.

Upregulation of these classical activation processes is accompanied in both WT1-positive subpopulations of aHSCs by downregulation of processes associated with quiescent cellular function.

### Mesothelial lineage cells re-engage a mesothelial signature

**Fig. 7:**
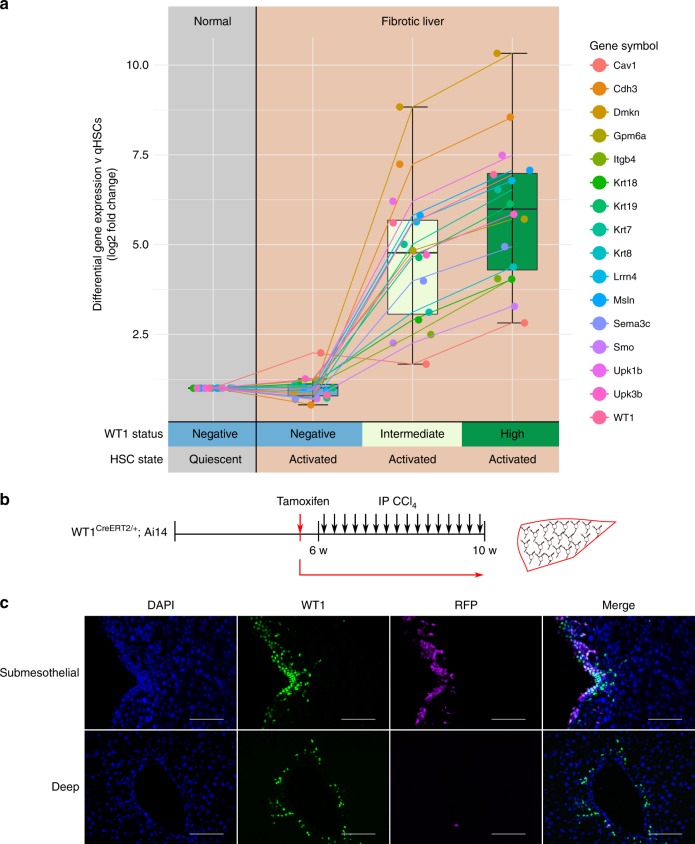
The hepatic mesothelium in development gives rise to a lineage of aHSCs that re-engages a mesothelial gene signature, and in adulthood is a limited de novo source of subcapsular WT1-defined aHSCs. **a** Only mesothelial-derived WT1-positive lineages of aHSCs re-engage a mesothelial gene signature after fibrotic injury. Data are represented as individual points with median (centre line), first and third quartiles (lower and upper box limits), 1.5x interquartile range (whiskers). **b** Lineage labelling of hepatic mesothelium was induced in adult WT1^CreERT2/+^;Ai14 animals before iterative injury with CCl_4_, and **c** livers stained for WT1 (green) and lineage-label (lilac). Scale bars 100 μm

The transcriptomic data from subpopulations of qHSCs and aHSCs were specifically interrogated to define the expression of a comprehensive, manually curated mesothelial-associated profile^20,29–33^ throughout their lineage (Fig. 7a). Strong expression of this gene expression signature was only present in WT1-intermediate and WT1-high aHSCs after injury and activation and expression was greater in the WT1-high population; these populations derive from precursor quiescent cells with ultimate origin from the embryonic mesothelium. WT1-negative aHSCs, selectively deriving from non-mesothelial derived quiescent precursors, do not engage this gene signature. There was no expression of mesothelial-associated genes in any qHSC population isolated from uninjured animals; this further indicates the absence of contamination of HSC isolates by non-buoyant mesothelial cells derived by density centrifugation.

### Mesothelium is the source of subcapsular WT1-positive aHSC

Previous reports have identified the adult hepatic mesothelium as a source of subcapsular aHSCs after injury^8^. The WT1^CreERT2/+^;Ai14 lineage tracing line was used to determine the WT1 status of this limited subcapsular population.

Lineage labelling was induced in adult male WT1^CreERT2/+^;Ai14 mice prior to chronic CCl_4_ injury (Fig. 7b). Activated HSCs were significantly more likely to be WT1-positive if they carried the lineage label reflective of mesothelial origin (Fig. 7c). 70.5 ± 4.7% of lineage labelled aHSCs were WT1-positive compared with 42.2 ± 5.9% of unlabelled aHSCs (Welch two-sample *t*-test, *t*(6.0798) = −5.1437, *p* = 0.002), confirming a specific relationship between mesothelial origin and WT1 status in adult injury, in addition to the equivalent relationship with mesothelial origin in development. WT1-positive aHSCs accounted for 45.8 ± 7.4% of the total aHSC population, a similar proportion to that observed in pericentral scars (10 pericentral fields/animal, *n* = 5 animals). Within the total subcapsular population of aHSCs, lineage-labelled cells accounted for only 5.0 ± 3.6% of the total population.

### Loss of *WT1* expression from aHSCs increases fibrosis

**Fig. 8:**
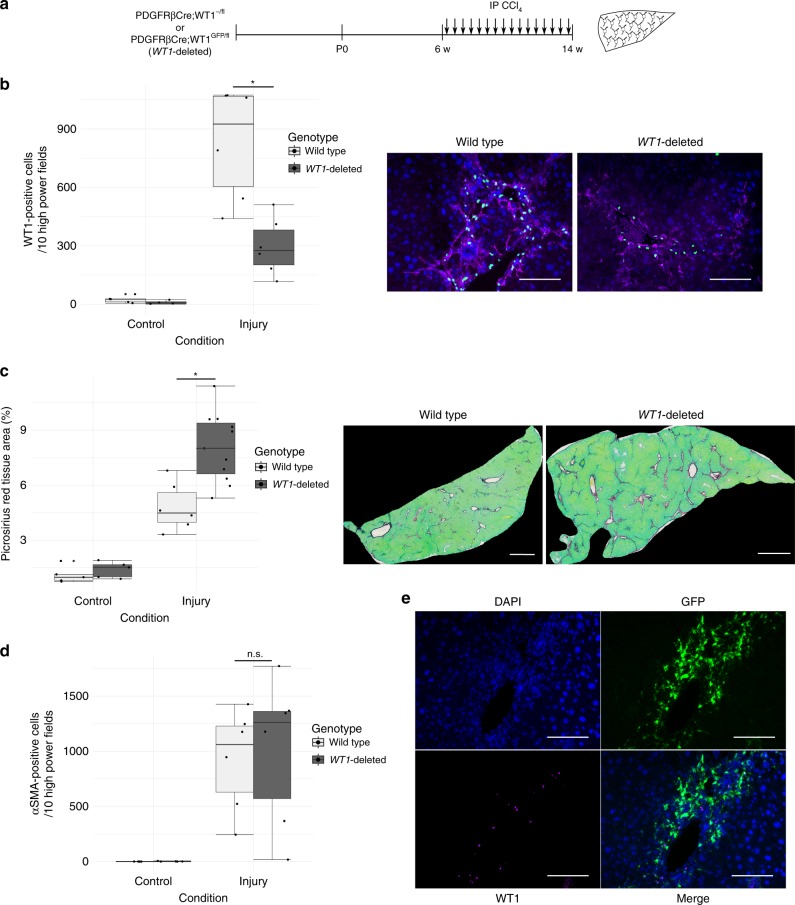
Loss of WT1 expression by PDGFRβ-expressing HSCs and myofibroblasts causes an enhanced fibrotic response to chronic injury without a change in myofibroblast number. **a** Chronic fibrosis was induced by iterative injury with CCl_4_ in animals in which WT1 had been constitutively deleted, or control animals. **b** The number of WT1-positive aHSCs after chronic injury in PDGFRβCre;WT1^−/fl^ animals is significantly reduced compared with wild-type animals (295.8 ± 59.4 vs 829.8 ± 116.3 WT1-positive cells/10 pericentral fields, *n* = 6; Welch two-sample *t*-test, *t*(7.4397) = −4.0896, *p = 0.00407). Example pericentral fields from the livers of injured wild-type and WT1-deleted animals stained for WT1 and αSMA are shown. Scale bars 100 μm. **c** There is an enhanced fibrotic response to chronic injury in WT1-deleted PDGFRβCre;WT1^−/fl^ animals (whole-slide PSR quantification of fibrotic matrix, 8.1 ± 0.6% vs 4.8 ± 0.5%, *n* = 6 (wild-type), *n* = 11 (PDGFRβCre;WT1^−/fl^); Welch two-sample *t*-test, *t*(13.765) = −4.194, *p = 0.0009388). Example PSR-stained sections of whole lobes of the livers of injured wild-type and WT1-deleted animals are shown. Scale bars 1 mm. **d** The enhanced fibrotic response to chronic injury in PDGFRβCre;WT1^−/fl^ animals is not associated with increased numbers of aHSCs compared with wild-type animals (1007.8 ± 273.8 vs 927.0 ± 186.5 αSMA-positive cells/10 pericentral fields; Welch two-sample *t*-test, *t*(8.819) = −0.244), *p* = 0.8128). Data are represented as individual points with median (centre line), first and third quartiles (lower and upper box limits), 1.5× interquartile range (whiskers). **e** The use of PDGFRβCre;WT1^GFP/fl^ animals allows continued GFP reporting to demonstrate the persistence of WT1-deleted aHSCs after pericentral chronic injury. Scale bars 100 μm

The functional role of *WT1* was determined by selective deletion from HSCs and MFBs using a constitutive PDGFRβCre line^34^ (Fig. 8a). Cohorts of wild-type (*n* = 6 animals) and PDGFRβCre;WT1^−/fl^ (*WT1*-deleted, *n* = 11 animals) male mice were chronically injured by CCl_4_ injection, and sections of liver stained for WT1.

**Fig. 9:**
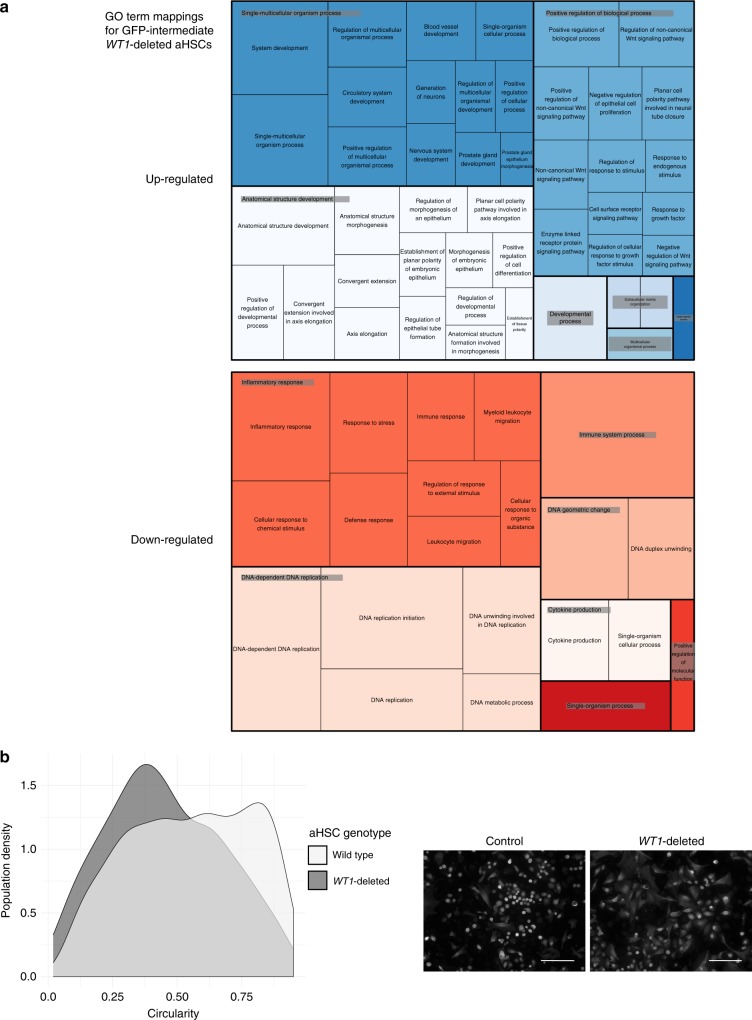
Loss of WT1 expression by aHSCs upregulates extracellular matrix and other profibrotic transcriptional pathways and permits transition to a myofibroblast morphology. **a** Genes mapping to GO terms for ‘extracellular matrix’, morphological and developmental transitions, non-canonical Wnt signalling and responses to growth factors are upregulated following WT1 loss in PDGFRβCre;WT1^−/fl^ animals after iterative injury with CCl_4_ in vivo. Genes mapping to immune and inflammatory responses and DNA replication are downregulated. **b** The population density distributions of circularity of activated RFP-positive cells under the condition of WT1 deletion was significantly different from that of control RFP(WT1)-positive cells (Bootstrapped two-sample Kolmogorov–Smirnov test, *p* = 0.002) during activation of qHSCs isolated from WT1^CreERT2/fl^;Ai14 animals in vitro, demonstrating a shift from round to myofibroblast-shaped aHSCs. Representative of three biological replicates. Scale bars 100 μm

There was a 64.3% reduction in the number of WT1-positive pericentral cells in *WT1*-deleted animals compared with wild-type animals (Fig. 9b, 295.8 ± 59.4 vs 829.8 ± 116.3 WT1-positive cells/10 pericentral fields, *n* = 6 animals; Welch two-sample *t*-test, *t*(7.4397) = −4.0896, *p* = 0.00407). The reduction in the number of WT1-positive aHSCs was accompanied by a 67.56% increase in fibrosis after injury (Fig. 8c, 8.1 ± 0.6% vs 4.8 ± 0.5% picro-sirius red (PSR) positivity; Welch two-sample *t*-test, *t*(13.765) = −4.194, *p* = 0.0009388). There was no difference in the number of scar-orchestrating αSMA-positive cells present (Fig. 8d, 1007.8 ± 273.8 vs 927.0 ± 186.5 αSMA-positive cells/10 pericentral fields; Welch two-sample *t*-test, *t*(8.819) = −0.244), *p* = 0.8128).

To determine if *WT1*-deleted cells persisted in the liver to coordinate enhanced fibrogenesis, a PDGFRβCre;WT1^GFP/fl^ line, in which GFP reporting from the *WT1* locus remained in *WT1*-deleted animals, was generated. A cohort of male PDGFRβCre;WT1^GFP/fl^ animals was injured by CCl_4_ injection for 8 weeks (*n* = 4 animals). 76.2 ± 3.6% of GFP-positive pericentral cells were WT1-negative (Fig. 8e); no cells were WT1 single positive (GFP-negative). This indicates the persistence of the *WT1*-deleted populations of MFBs during enhanced fibrogenesis.

### *WT1* loss enhances fibrotic HSC transcription and transition

To allow examination of residual MFBs in *WT1*-deleted animals from which *WT1* expression had been lost, an additional Cre reporter line was generated (PDGFRβCre;WT1^GFP/fl^;Ai14). Male PDGFRβCre;WT1^GFP/fl^;Ai14 (labelled *WT1*-deleted) and control PDGFRβCre;WT1^GFP/+^;Ai14 animals were chronically injured by CCl_4_ injection (*n* = 6 animals). Quiescent and activated HSCs were isolated the day after the final injection by density centrifugation. Cre reporter (RFP) positive GFP-high and GFP-intermediate aHSCs were obtained by FACS from both labelled *WT1*-deleted and control animals and used for RNAseq analysis.

In GFP-high aHSCs, 40 genes were upregulated after *WT1* deletion compared with only ten that were downregulated (Supplementary Data 8). Upregulated genes mapped to the GO terms ‘proteinaceous extracellular matrix’ and ‘extracellular region’; the limited number of downregulated genes did not map significantly to any GO term.

In GFP-intermediate aHSCs, 155 genes were upregulated and 170 downregulated after *WT1* deletion (Supplementary Data 9). For those genes that were upregulated in GFP-intermediate cells, GO terms ‘extracellular matrix organisation’ and ‘extracellular region’, terms for positive regulation of non-canonical Wnt signalling, responses to growth factors and cell surface receptors, and morphological cellular and developmental transitions were represented (Fig. 9a). In contrast, downregulated genes mapped to GO terms relating to immune and inflammatory responses, and DNA replication.

This suite of responses to *WT1* loss suggests two broad roles for *WT1* in aHSCs in fibrotic disease. Upregulation of ‘extracellular matrix organisation’, ‘responses to extracellular stimuli’, and ‘non-canonical Wnt signalling’ after *WT1* loss suggests that WT1 has a tonic inhibitory role that limits fibrogenesis during injury. The most downregulated gene in GFP-intermediate cells after WT1-deletion was Ctrb1 (log fold-change −9.35, adjusted *p*-value 0.016). Downregulation of immune and inflammatory functions in the GFP-intermediate subpopulation, the same functions overrepresented in WT1-intermediate compared with WT1-high aHSCs, indicates a critical role for WT1 orchestrating these additional functions of aHSCs not directly related to scarring.

The upregulation genes mapping to morphological cellular and developmental transitions in the *WT1*-deleted GFP-intermediate subpopulation merited morphological evaluation of aHSCs. Morphology of aHSCs could not be reliably quantitated in situ after in vivo injury, so *WT1* deletion during in vitro activation was examined. qHSCs were isolated from uninjured WT1^CreERT2/fl^;Ai14 animals (where Cre mediated excision of the single floxed allele completely prevents *WT1* expression), and the morphology of RFP (Cre-reporter) aHSCs examined after 7 days under the condition of *WT1* deletion. Control cultures derived from WT1^CreERT2/+^;Ai14 animals, where tamoxifen addition induces RFP expression alone, were used.

The mean circularity of all RFP-positive aHSCs reduced by 17.5% with *WT1* deletion (from 0.55 ± 0.01 to 0.45 ± 0.01). The population density distributions of circularity of activated RFP-positive cells with *WT1* deletion was significantly different from that of control RFP(WT1)-positive cells (Fig. 9b, bootstrapped two-sample Kolmogorov–Smirnov test, *p* = 0.002, 345 cells from representative experiment of three independent cell preparations). The demonstrable transition from a dominant round morphology in WT1-positive aHSCs to MFB-shaped cells after *WT1*-deletion is consistent with *WT1* maintaining the round morphology associated with reduced fibrogenic capacity in WT1-high cells during injury.

## Discussion

Activation of qHSCs is the dominant process generating scar-orchestrating cells during fibrotic liver injury^1^. We have demonstrated profound transcriptional, morphological, and fibro-spatial heterogeneity of aHSCs after injury, defined by *WT1* expression. Recognising and understanding the functional significance of aHSC heterogeneity will inform more nuanced understanding of fibrogenesis, akin to heterogeneity in perivascular cells in injury defined in other systems^35^. For example, injury with CCl_4_ is pericentral, and scarring develops radially with continued damage; WT1-positive populations of aHSCs are rapidly evident after injury and are most closely apposed to central veins, so may represent a specific initiator population of fibrogenic cells.

Separate populations of aHSCs, defined by *WT1* expression, have distinct transcriptional profiles and fibrogenic capacities. The WT1-high population is rounded whilst the WT1-intermediate, most fibrogenic, population is classical MFB-shaped. Deletion of WT1 promoted the formation of these classical MFBs, with enhanced expression of genes associated with developmental and cellular transitions, suggesting that *WT1* may be controlling population plasticity and that high WT1 levels maintain the less fibrogenic phenotype.

We have also demonstrated that precursor qHSCs in the uninjured adult liver are members of distinct lineages, dependent upon mesothelial or non-mesothelial developmental origin, and offering a developmental basis for previously recognised heterogeneity^36,37^. Further, lineage membership defines discrete transcriptomic profiles suggesting significant functional differences. The recognition of population diversity based upon developmental lineage is important to drive investigation of HSC biology; potential functions attributed to qHSCs can be segregated based upon origin, with mesothelial-derived qHSCs having upregulated GO terms indicating functions relating to vasculature regulation and qHSCs of non-mesothelial origin having GO term upregulation for immune and injury response-related functions. Furthermore, the heterogenous qHSC populations are complementary in their collagen contribution to the space of Disse. Mesothelial derivation of a subpopulation of qHSCs is analogous to the mesothelial origin of a proportion of visceral white adipose tissue^18^, indicating that mesothelial origin is a common source of functionally significant heterogeneity in mesothelial-covered organs^15,38^.

**Fig. 10:**
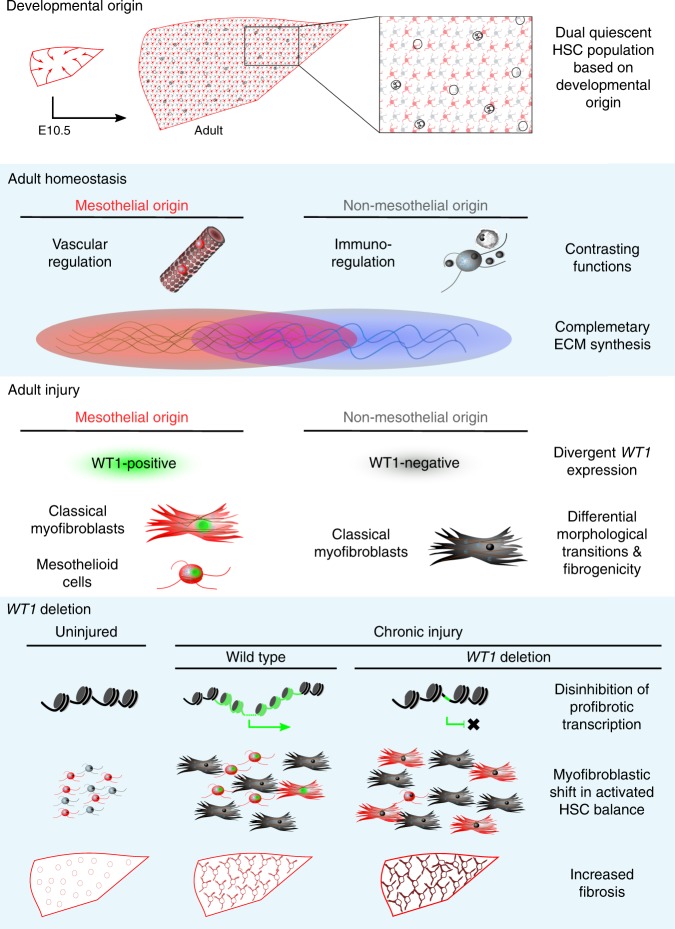
WT1 is a paradoxical negative regulator of fibrogenesis in distinct subpopulations of aHSCs defined by WT1 expression. WT1-positive subpopulations in liver injury are generated by activation of specific progenitors that derive from the embryonic hepatic mesothelium in early development. Developmental origin determines the functional categorisation of qHSCs; those derived from the mesothelium demonstrate engagement of vascular regulatory pathways, whilst those from non-mesothelial sources have immunomodulatory transcriptional profiles

Critically, these previously unrecognised subpopulations of HSCs in the homoeostatic and fibrotic liver can be defined as part of a complete discreet lineage (Fig. 10); WT1-positive scar-orchestrating cells derive from a qHSC population that is enhanced for pericyte functions and whose ultimate origin is the hepatic mesothelium during development. Identification of mesothelial-derived qHSCs throughout lobules in homoeostasis and WT1-positive scar-orchestrating cells in all models and human diseases indicates that mesothelial origin of distinct quiescent precursors capable of generating defined subpopulations of scar-orchestrating cells is an injury-agnostic, fundamental feature of hepatic fibrogenesis. These relationships between embryonic mesothelial origin and heterogeneous populations of quiescent progenitors and scar-orchestrating cells may be shared in other mesothelial covered organs, particularly the pancreas. Obviously, we have defined the complete cellular lineage of half of the scar-orchestrating cells after liver injury; the possible heterogeneity and alternative origin(s) of the WT1-negative cells have not been sought.

Categorical demonstration that pericentral aHSCs after injury, present deep within the parenchyma and without mesothelial contamination, express a signature of genes previously identified in the mesothelium identifies a unique inter-generational signifier. Only the populations whose ancestry traces to the embryonic mesothelium express this transcriptional signature and its re-engagement reflects their lineage. The biphasic morphology evident in these activated adult cells is akin to that seen in mesothelial malignancy (mesothelioma) or some forms of Wilms’ tumour. This suggests that *WT1*-related morphological plasticity is not limited to malignant disease but occurs in scarring after injury in situations where a mesothelial origin for scar-orchestrating can be identified. Whilst a shared microarchitectural location and buoyancy reflective of lipid storage merits designation of both populations as quiescent hepatic stellate under current dogma, the differences in developmental origin, quiescent transcriptomic profile, and phenotype in response to injury of the lineages raise the possibility that these could legitimately be considered to be completely different cell types.

Deletion of *WT1* from PDGFRβ-positive HSCs did not lead to cell loss; instead, persisting subpopulations orchestrated enhanced fibrogenesis. The associated change in transcriptional profiles after deletion were those of enhanced growth factor responsiveness and non-canonical Wnt signalling rather than purely upregulation of ECM gene expression. The most downregulated gene after WT1 loss was Ctrb1; in addition to documented matrix degradative capacity^39^, known and predicted protein–protein interactions of chymotrypsinogen B1 documented by the STRING database^40^ are dominated by interactions with matrix metalloproteinases, serine proteases, and peptidase inhibitors (Supplementary Fig. 6). Given the significantly enhanced scarring after WT1 deletion, this suggests that Wnt-signalling has a critical role driving HSC scarring capacity, requiring suppression by WT1 and that chymotrypsinogen B1 could be explored as a central node in the network of post-translational modifiers of matrix accumulation and remodelling.

Mesothelium, as the name indicates, is characterised by both mesenchymal and epithelial properties, strongly expresses WT1, and is increasingly recognised as a highly specialised tissue. WT1 has a well-defined role in controlling morphological transitions. In the developing kidney, WT1 is required for nephron formation by regulating mesenchymal–epithelial transition^14^. Conversely, WT1 is required for the formation of mesenchymal lineages in the heart by epithelial–mesenchymal transition of the epicardium^12^. The epicardium is the cardiac mesothelium, so this may be better defined as mesothelial–mesenchymal transition.

In the classical model of hepatic fibrogenesis, morphological plasticity of HSCs is accepted during activation. More profound HSC plasticity in the liver is more contentious^41^. Although there is evidence to indicate epithelial regenerative capacity after injury by formal mesenchymal–epithelial HSC transition^42–45^, with Hedgehog signalling central to this, and the formation of HSCs by epithelial–mesenchymal transition of hepatic epithelia, other studies do not support such transitions^46,47^.

Given the precedent of WT1 regulation of epithelial-mesenchymal transition and mesenchymal-epithelial transition, and the role of the mesothelium in this, it is important to explicitly evaluate our findings in the context of such transitions. We have demonstrated significant punctuated and bidirectional morphological plasticity throughout the complete temporal extent of the mesothelial-derived HSC lineage, supporting the capacity for extreme flexibility by this HSC lineage suggested by earlier work. At the point of developmental origin of the lineage there is mesothelial–mesenchymal transition^10^. Subsequent responses to adult injury could be interpreted as a partial reversal of this by mesenchymal–mesothelial transition, indicated by both the re-engagement of a WT1-high mesothelioid gene signature and the acquisition of a characteristic ‘rounded’ morphology; a classical (‘activated’) scar-orchestrating transcriptional profile is also acquired. At the same time a distinct, more fibrogenic, population of aHSCs within the same lineage re-engages the mesothelial gene profile to a lower level and demonstrates a classical MFB shape. The WT1-high population after adult injury expresses the high levels of the *Smo*, the regulator of HSC transitions defined by the study of Michelotti et al.^42^, as part of the re-engagement of a mesothelial signature. Finally, whilst loss of expression of WT1 by HSCs did not change the expression of the mesothelial gene signature, including *Smo*, it permitted greater scar formation through enhanced aHSC fibrogenic transcriptional profile expression but no change in cell number. This was associated with a morphological shift to favour the myofibroblastic morphology, a finding that could be considered a failure of scar-mitigating mesenchymal–mesothelial transition during injury or as a final, aberrant mesothelial-MFB transition.

Intrinsic profibrotic factors induced by activation in HSCs by injury are well described, and antifibrotic intrinsic factors present within qHSCs have been observed to diminish during injury^48^. However, *WT1* represents a new paradoxical category of intrinsic regulatory factors, one that is antifibrotic but induced only in injury; this may permit additional means of fine-tuning of the fibrotic response.

## Methods

### Murine line details and generation

The animals used for injury modelling in this study were all on a C57Bl/6 background aged within 8–12 weeks old and >20 g body weight at the start of the experiments. Male mice were used for in vivo studies; male and female mice were used for in vitro studies. Animals were housed in a specific pathogen-free environment and kept under standard conditions with a 12 h day/night cycle and access to food and water ad libitum. All animal experiments were carried out under procedural guidelines, severity protocols and with ethical approval from the University of Edinburgh Animal Welfare and Ethical Review Body (AWERB) and the Home Office (UK).

Separate power calculations were not routinely performed; however, animal numbers were chosen to reflect the expected magnitude of response taking into account the variability observed in previous experiments.

For mechanistic experiments where a change in fibrotic response was expected, a power calculation using the anticipated efficacy of modification was used. An assumption of a 30% change in collagen content between transgenic and normal animals was used, with an 80% chance of detecting this expected difference accepted; using our previous data regarding population distribution and effect size, and for a two-sided significance level of 5% and power of 80%, at least six animals per group were required to compare normal versus knockout/transgenic mice where fibrosis is quantified by PSR staining.

WT1^GFP/+^ knockin reporter mice were originally provided by Sugiyama^17^ (Osaka University School of Medicine, Japan).

WT1^CreERT2/+^;Ai14 mice for lineage tracing were created by crossing knockin mice expressing tamoxifen-inducible Cre recombinase at the *WT1* promoter locus^18^ (WT1^CreERT2/+^) with the Ai14 Cre reporter^19^. In this reporter line, a construct containing the CAG promoter followed by flox-STOP-flox controlled tdTomato has been inserted into the wild-type Rosa26 locus. In the WT1^CreERT2/+^;Ai14 line, Cre-mediated excision of the stop codon allows cytoplasmic tdTomato expression in WT1-positive cells and their progeny, after induction with 4-hydroxytamoxifen.

The *WT1*-conditional background (WT1^fl/fl^)^12^ was used to create lines allowing *WT1* deletion. For selective deletion of *WT1* from qHSCs, the constitutive PDGFRβCre line was obtained from Henderson et al.^34^. PDGFRβCre males were crossed with WT1^fl/fl^ females to create PDGFRβCre;WT1^+/fl^ animals; there is germline PDGFRβ expression such that only the *WT1* null allele is transmitted. To generate PDGFRβCre;WT1^−/fl^ animals, PDGFRβCre;WT1^+/fl^ males were crossed with WT1f^l/fl^ females.

To allow identification and isolation of persisting aHSCs showing loss of expected *WT1* expression, PDGFRβCre;WT1^GFP/+^ males were crossed with WT1^fl/fl^ females to generate the PDGFRβCre;WT1^GFP/fl^ line; the control PDGFRβCre;WT1^GFP/+^;Ai14 line was generated by crossing PDGFRβCre;WT1^GFP/+^ males were crossed with WT1^+/+^;Ai14 females.

Finally, for inducible deletion of *WT1* during in vitro activation, male WT1^CreERT2/+^;Ai14 animals were crossed with WT1^fl/fl^ females to generate the WT1^CreERT2/fl^;Ai14 line.

### Chronic fibrotic in vivo injury models

Liver fibrosis was induced in cohorts of male mice by 4 or 8 weeks CCl_4_ injection twice weekly, 0.25 µl g^−1^ body weight in a 1:3 ratio with sterile olive oil^49^, or vehicle alone. CCl_4_ undergoes conversion to the CCl_3_ radical in pericentral hepatocytes, and CCl_3_ initiates lipid peroxidation leading to hepatocellular death. The resultant inflammation leads to activation of HSCs and the development of pericentral fibrosis with spur formation and early central–central bridging fibrosis over this period. Mice of the appropriate genotype were randomly allocated to olive oil or carbon tetrachloride injection as they became suitable for model participation. Blinding to control or injury groups was not possible as the injury is macroscopically and microscopically apparent.

WT1^GFP/+^ mice were injured for 8 weeks and culled 1 day after the final injection for isolation of HSC populations by flow cytometry (*n* = 3) or 3 days after for histological examination (*n* = 6).

Sections of formalin-fixed paraffin-embedded fibrotic liver from wild-type mice generated by alternative models of liver fibrosis with complementary histological patterns of injury were obtained (*n* = 3 for each model). BDL and DDC^50^ dietary injury cause bile duct obstruction leading to inflammatory portal and periportal injury and fibrosis, modelling destructive peripheral cholangiopathies. TAA administration^51^ induces both periportal and pericentral fibrosis to model diseases with portal, interface and lobular hepatitis (e.g. Autoimmune Hepatitis (AIH)).

### Acute CCl_4_ injury in vivo

Acute injury causing de novo activation of qHSCs was induced in wild-type animals by a single intraperitoneal injection of CCl_4_ (0.25 μl g^−1^ or 1 μl g^−1^ body weight) or olive oil vehicle, as described^34^. Cohorts of animals were culled the day following injury to allow determination of the induced hepatocellular necrosis (*n* = 4, 0.25 µl g^−1^; *n* = 3, 1 µl g^−1^). To quantify WT1-positive cell number following injury, cohorts injured with an injection of 0.25 µl g^−1^ CCl_4_ were culled 3 and 7 days after injury (*n* = 3), and cohort injured with 1 µl g^−1^ CCl_4_ or vehicle control culled 3 days after injury (*n* = 3). Animals were not randomised to injury or control groups. Blinding to control or injury groups was not possible as the injury is macroscopically and microscopically apparent.

### Lineage labelling in vivo

Nuclear Cre translocation and labelling of WT1-positive cells were induced in adult WT1^CreERT2/+^;A14 mice by 5 consecutive days of tamoxifen IP injections (100 mg kg^−1^). To trace the contribution of the mesothelium to the adult HSC population in the absence of injury, cohorts of female animals were culled without injury up to 45 days after labelling (*n* = 3/time point). To determine the contribution of the adult hepatic mesothelium to the *WT1*-defined subpopulations of aHSCs, a cohort of male animals was used for 4 weeks of chronic fibrotic injury with CCl_4_, beginning on the final day of tamoxifen administration (*n* = 5). No labelling is evident without tamoxifen injection. Blinding to tamoxifen administration status is not possible as Ai14 label is macroscopically apparent.

To generate developmentally induced *WT1* lineage-labelled HSCs, male WT1^CreERT2/+^;Ai14 mice were crossed with wild-type female mice. Pregnant females received a single gavaged dose of tamoxifen (200 mg kg^−1^) at E10.5. Pups were delivered by Caesarean section at E18.5 and fostered onto a wild-type post-partum female. Male lineage-labelled animals were used for chronic fibrotic injury with CCl_4_ (*n* = 5), and female lineage-labelled animals used for examination of lineage-labelled populations with immunofluorescence (*n* = 3) or isolation of labelled and unlabelled qHSCs for RNAseq (*n* = 3). No labelling is evident without maternal tamoxifen administration. Blinding to tamoxifen administration status is not possible as Ai14 label is macroscopically apparent.

### In vivo surface mesothelial labelling

Surface mesothelium was labelled by the intraperitoneal injection of a single dose of a non-toxic fluorescent surface dye (CellTracker Red CMTPX, Thermo Fisher Scientific), as per manufacturer’s instruction (1:1000 dilution of 10 mM stock solution in sterile PBS, 5 μl g^−1^ body weight) for 60 min before sacrifice.

### Human explant studies

Human tissue was obtained by approved application to the Lothian NRS Human Annotated Bioresource that is authorised to provide unconsented anonymised tissue under ethical approval number 15/ES/0094 from the East of Scotland Research Ethics Service REC 1. Sections of formalin-fixed paraffin-embedded explant fibrotic human liver from the deep right lobe, removed at the time of orthotopic liver transplantation, were received from eight cases (two cases of alcoholic liver disease, two cases of primary biliary cirrhosis, two cases of cryptogenic cirrhosis, and one each of chronic viral hepatitis (HCV), and primary sclerosing cholangitis). Explant livers were received fresh on ice and fixed immediately by the installation of 4% v/v neutral buffered formalin (Genta medical, UK) via the hepatic veins. All tissue was from cases from 2006 onwards and received anonymised to all details other than aetiology.

### Histology and quantitative pathology

Samples of each lobe of livers were immediately formalin-fixed before paraffin-embedding in a single block to allow histological examination. Haematoxylin-and-eosin-stained sections were prepared from each animal. PSR-stained sections were prepared^52^ and whole-slide images acquired with Dotslide VS-ASW software using a motorised stage and an Olympus BX51 microscope, using an Olympus PlanApo 2X lens and Olympus XC10 camera. The PSR-positive proportion of all tissue from each whole-slide image was determined after applying the same colour threshold to all images as a script within the FIJI^53^ implementation of ImageJ2^54^.

For immunofluorescence, antigen retrieval was achieved by microwaving in Tris-EDTA pH 9.0. Endogenous peroxidase activity was blocked by 3% (v/v in phosphate-buffered saline) H_2_O_2_ pretreatment. Primary antibodies at appropriate dilutions were applied and slides incubated at room temperature for 1 h or 4 °C overnight. Details of primary antibodies and dilutions are given in Supplementary Table 9. Negative controls were performed using identical concentrations of species and isotype-matched non-immune immunoglobulin in place of primary antibody (where details were available) or omission of primary antibody.

To determine fibro-spatial data from individual cells after CCl_4_ injury, 10 pericentral fields (×20 objective lens) were acquired using a Zeiss Axioplan II microscope and Photometrics CoolSNAP HQ2 camera (*n* = 6). Nuclear location of cells and central vein outlines were marked for each in FIJI, and points of interest imported into R. The nearest point to the central vein of each cell was determined using *spatstat*^55^. Population distributions based on distance to central vein were assessed by a bootstrapped version of the Kolmogorov–Smirnov test using the *Matching* package^56^.

For confocal colocalization of HSC markers with WT1 or GFP after CCl_4_ injury, images were acquired from a total of 10 pericentral fields from three biological replicates using a Nikon A1R confocal microscope.

To histologically quantify necrosis after acute injury, slides were scanned to create a single image with Dotslide VS-ASW software using a motorised stage and an Olympus BX51 microscope, acquiring images using an Olympus PlanApo 2X lens and Olympus XC10 camera. Images were analysed using the Trainable Segmentation plugin^57^ implementing WEKA^58^ in FIJI. A separate classifier identifying necrotic and viable tissue was determined and applied to all tissue in each image.

### Flow cytometry analysis and sorting

Analysis and sorting of HSCs derived from WT1^GFP/+^, WT1^CreERT2/+^;Ai14, PDGFRβCre;WT1^GFP/fl^, and PDGFRβCre;WT1^GFP/+^;Ai14 animals was undertaken using a FACSAria (BD). Freshly isolated cells from injured or uninjured liver were used after red cell lysis. Data were analysed using FlowJo. For in vitro activation studies, data were collected from 12 independent cell preparations; sorting into separate subpopulations defined by *WT1* expression was undertaken directly for RNA preparation (*n* = 5) or for further culture and morphological studies (*n* = 8); cells from one preparation were sorted for both purposes on one occasion. Sorting based on single channel WT1^GFP^ expression was as documented in the relevant plots; strategies for Ai14 Cre-reporter lines are shown in Supplementary Fig. 7.

### Hepatic stellate cell isolation and in vitro studies

Quiescent and activated HSCs were isolated, after digestion, by density centrifugation as described^59^. qHSCs isolated from uninjured livers were activated by culture on plastic, or culture on sterile glass coverslips to allow subsequent immunofluorescent staining.

RFP expression was induced in aHSCs from WT1^CreERT2/+^;Ai14 animals at day 6 of culture on glass coverslips by the addition of 1:1000 1 mM 4-hydroxytamoxifen in ethanol to cultures. Cultures were examined by inverted fluorescent microscopy after 24 h to visualise native RFP expression, and then fixed in ethanol:methanol 1:1 for 10 min at −20 °C before immunofluorescent staining.

For in vitro deletion of Wt1 from aHSCs during activation, isolated qHSCs from WT1^CreERT2/fl^;Ai14 animals and WT1^CreERT2/+^;Ai14 controls were cultured to day 7 in the presence of 1:1000 1 mM 4-hydroxytamoxifen in ethanol.

### In vitro morphometric analysis

qHSCs from uninjured WT1^GFP/+^ animals were activated by 7 days of culture on plastic, and subpopulations defined by Wt1(GFP) expression obtained by FACS. Subpopulations were plated separately back onto plastic and allowed to adhere overnight. Cells were labelled by addition of a non-toxic fluorescent surface dye (CellTracker Red CMTPX, Thermo Fisher Scientific), as per manufacturer’s instruction, and examined by inverted fluorescent microscopy after 30 min using the EVOS FL Cell Imaging System (Thermo Fisher Scientific). For subpopulation, five low-power images (×4 objective) were acquired. ‘Circularity’ of individual cells (4*π*(area perimeter^−2^)) was determined using in FIJI.

Native RFP expression was used in place of the surface dye for morphometric analysis of WT1^CreERT2/fl^;Ai14 and WT1^CreERT2/+^;Ai14 aHSCs.

### Transcriptomics

Cells were sorted by flow cytometry directly into RLT (Qiagen) containing β-ME to maintain sample integrity. RNA was isolated using RNeasy Micro Kit (Qiagen).

For microarray data, whole-genome expression was determined with the Illumina MouseWG-6 v2.0 Expression BeadChip after RNA preparation with the Ambion Illumina TotalPrep RNA amplification kit. Unnormalised summary probe profiles were exported from BeadStudio to tab-delimited text files and read into the R environment for analysis with Bioconductor^60^ package *limma*^61^. Expression data were background corrected using negative controls, then quantile normalised and finally log2 transformed. Differentially expressed probes were determined by fitting a linear model, and probes determined as up- or downregulated by *decideTests* using the default arguments of adjustment of the *p*-value for multiple comparisons (Benjamini and Hochberg method^62^) and an adjusted *p*-value threshold of 0.05.

RNAseq was undertaken by GATC Biotech on an Illumina HiSeq. Fastq files were mapped with bowtie 2^63^, and differential expression for subsequent GO and SPIA analysis was determined by Cuffdiff2^64^, using an adjusted *p*-value threshold of 0.05. Differential expression for PCA was determined using DESeq2^65^.

GO analysis for was undertaken with *GOsummaries*^66^ or Revigo (for knockout experiments)^67^, using g:Profiler^68^ to ensure current Ensembl annotations are used. Chord diagrams of differentially expressed genes related to specific GO annotations were created using *GOplot*^69^. Pathway analysis was undertaken with *SPIA*^70^, after updating KEGG pathway^71^ annotations (October 2016). SPIA combines enrichment analysis with a measure of the actual pathway perturbation under a given condition based on pathway topology, calculating a global pathway significance *p*-value combining the enrichment and perturbation *p*-values.

### Quantitative PCR

For the determination of gene expression by qPCR (undertaken using an Applied Biosystems HT7900) on isolated cell populations, the Universal ProbeLibrary (Roche) was used, and primers designed using the ProbeFinder web-based tool based on Primer3^72^. Sequences and probe numbers are shown in Supplementary Table 10.

### Quantification and statistical analysis

The RStudio implementation of R was used for all statistical analysis. Data are presented as mean ± s.e.m. All group sizes (*n*) refer to biological replicates, either individual animals for in vivo studies or independent cell isolations for in vitro studies. Validation qPCR was undertaken using triplicate technical replicates for each biological replicate.

Prior to testing, the normality of data was determined using Shapiro–Wilk testing and by examination of qq plots. For comparison of continuous variables between two groups after assumptions of normality were satisfied, the Welch (unequal variance) *t*-test was used^73^. For a comparison between more than two groups, a one-way ANOVA with post-hoc Tukey was used. Distributions of measured features of subpopulations from single animals were compared with a bootstrap version of the Kolmogorov–Smirnov test^56^. To determine the correlation between two variables, two-sided Spearman’s rank correlation coefficients were calculated.

### Reporting summary

Further information on research design is available in the Nature Research Reporting Summary.

## Supplementary information

Supplementary Information

Peer Review

Description of Additional Supplementary Files

Supplementary Data 1

Supplementary Data 2

Supplementary Data 3

Supplementary Data 4

Supplementary Data 5

Supplementary Data 6

Supplementary Data 7

Supplementary Data 8

Supplementary Data 9

Reporting summary

## Data Availability

The RNAseq and microarray datasets generated and analysed in the study have been deposited in the Gene Expression Omnibus (GEO), as accession number GSE103108. The authors declare that all other relevant data supporting the findings of this study are available within the Article and its Supplementary Information files, or from the corresponding author on reasonable request. A reporting summary for this article is available as a Supplementary Information file.
