# Supplementary material for: Embryonic mesothelial-derived hepatic lineage of quiescent and heterogenous scar-orchestrating cells defined but suppressed by WT1
Source: Nat Commun. 2019 Oct 15;10:4688. doi: 10.1038/s41467-019-12701-9 (PMC6794268; doi:10.1038/s41467-019-12701-9)
Supplement: Supplementary file 1 — Supplementary Information [file 41467_2019_12701_MOESM1_ESM.pdf]

Supplementary Information

Embryonic mesothelial-derived hepatic lineage of quiescent and heterogenous scar-orchestrating cells defined but suppressed by WT1

Kendall et al

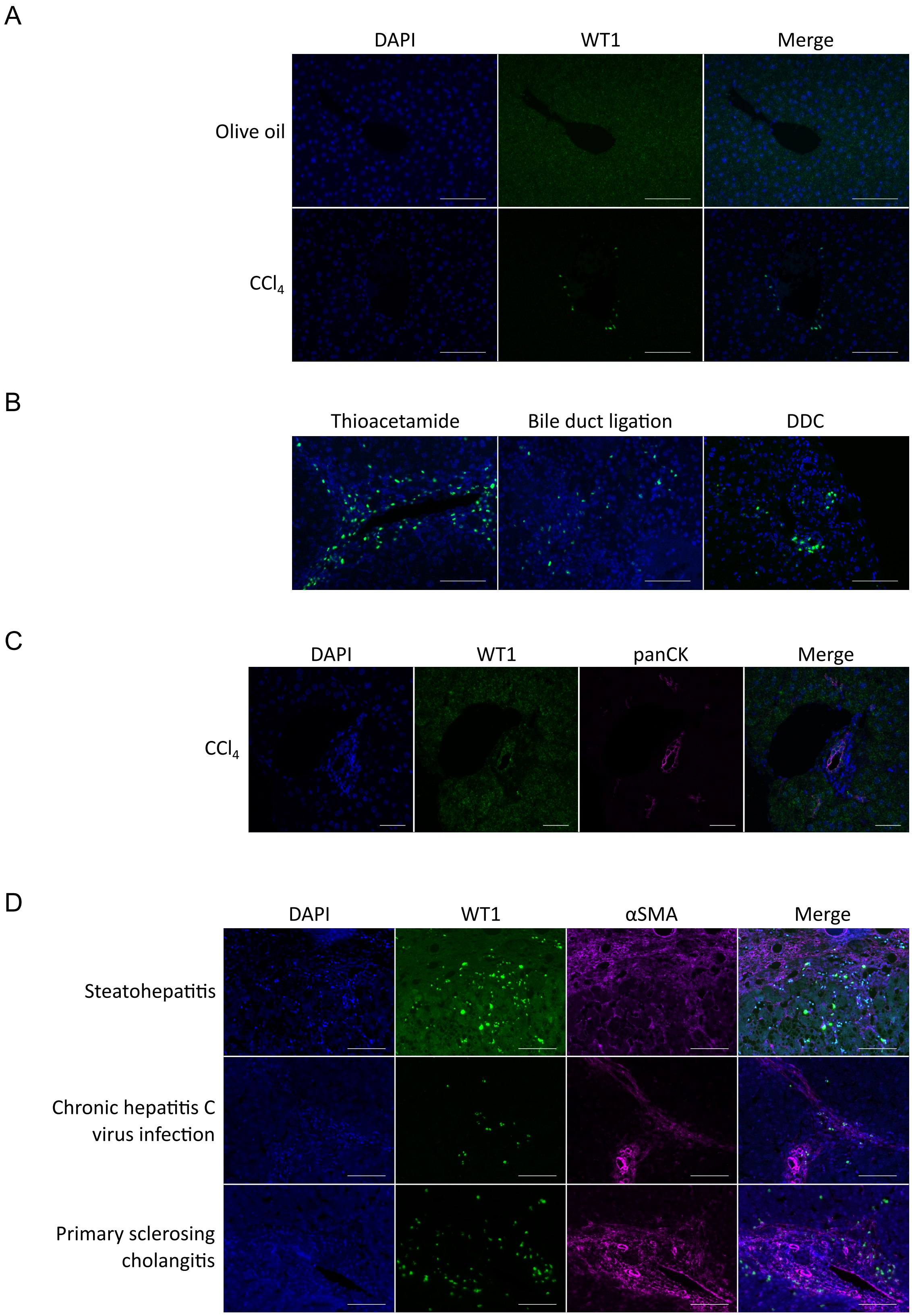

Supplementary Figure 1. WT1-positive activated hepatic stellate cells are found in a spectrum of murine models and human fibrotic disease. (A) No WT1-positive cells are present in lobules of uninjured (quiescent) control animals, injected with vehicle-only control; pericentral WT1-positive cells are present from 3 days after a single injection of CCl_4. Scale bars 100 μm (green, WT1). (B) WT1-positive cells are present after chronic injury in murine models using thioacetamide administration, bile duct ligation, and 3,5-diethoxycarbonyl-1,4-dihydrocollidine (DDC) diet. (green, WT1). (C) No WT1-positive cells are present in epithelial cells or other cells within or adjacent to portal tracts after injury with CCl_4 for 9 weeks (green, WT1; lilac, cytokeratin). Scale bars 200 μm. (D) WT1-positive myofibroblasts are present in human cirrhotic explant hepatectomies for steatohepatitis (alcoholic liver disease), chronic biliary disease (primary sclerosing cholangitis), and lobular hepatitis (chronic hepatitis C virus infection). Scale bars 100 μm (green, WT1; lilac, αSMA).

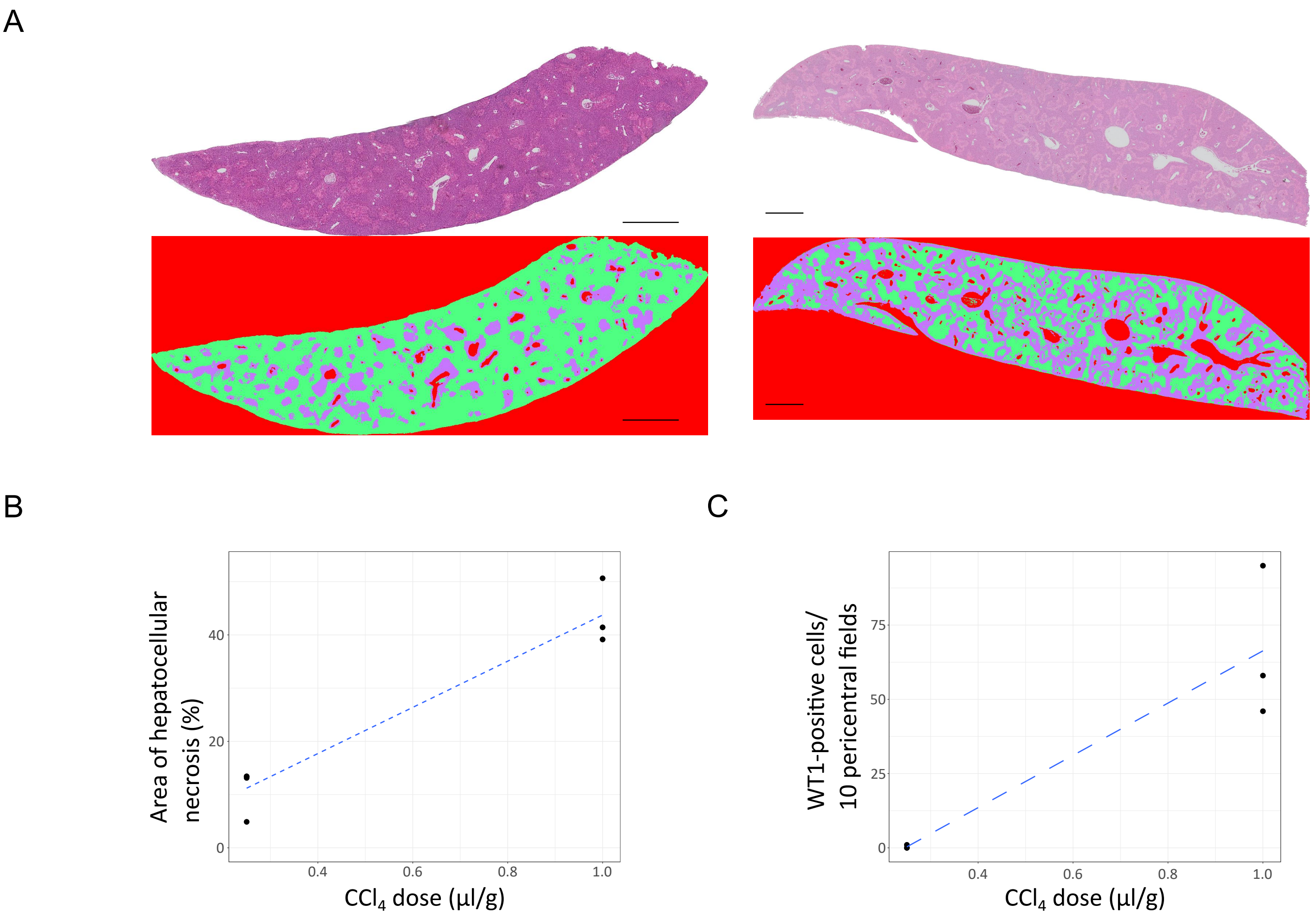

Supplementary Figure 2. Larger doses of CCl_4 produce more parenchymal necrosis and larger numbers of WT1-positive cells. (A) A larger single dose of CCl_4 produced more pericentral hepatocellular necrosis (left – 0.25 µl/g, right – 1 µl/g, representative haematoxylin and eosin stained images of liver and paired classified image; lilac, necrosis; green, viable; red, space/lumen; scale bars 1 mm). (B) Strong positive correlation between the size of the single injected dose of CCl_4 and the extent of hepatocellular necrosis (Spearman's rank correlation r_s=0.866, p=0.01172). (C) Strong positive correlation between the size of injected dose and number of WT1-positive cells (Spearman's rank correlation r_s=0.8911, p=0.01713).

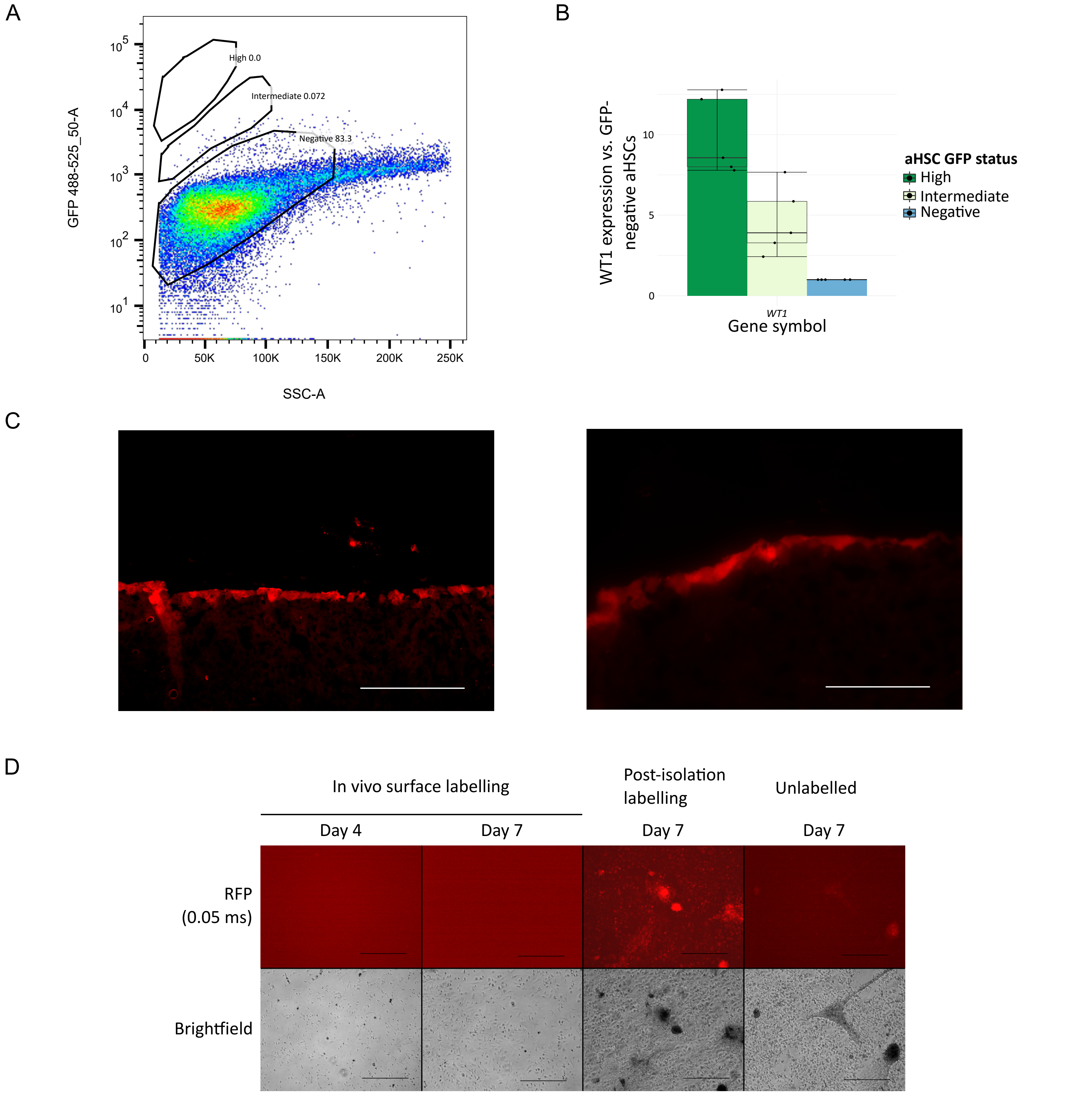

Supplementary Figure 3. GFP intensity in $WT1^{GFP/+}$ cells matches WT1 expression and neither WT1-positive cells nor contaminating surface mesothelial cells are not present in primary preparations of HSCs by density centrifugation. (A) The buoyant HSC fraction isolated from uninjured $WT1^{GFP/+}$ animals and analysed before activation by culture on plastic contains minimal WT1-positive cells (represent. (B) QPCR for WT1 from subpopulations of aHSCs from $WT1^{GFP/+}$ animals, activated in vitro and isolated by flow cytometry based on GFP intensity, confirmed a strong correlation between WT1 expression and GFP intensity (Spearman's rank correlation r_s=0.962, p=$1.009x10^{-8}$, n=5). Data are represented as individual points with median (centre line), first and third quartiles (lower and upper box limits), 1.5x interquartile range (whiskers). (C) Animals received an intraperitoneal injection of CellTracker solution to label the hepatic mesothelium. Frozen sections demonstrate efficient surface mesothelial labelling. Scale bars 100 µm (left) and 50 µm (right). (D) No labelled contaminating mesothelial cells were present in cultures of isolated qHSCs during seven days of culture activation. Positive control labelling of isolated qHSCs before plating demonstrates label persistence throughout activation. All fluorescent images acquired at 0.05 ms exposure. Scale bars 200 µm.

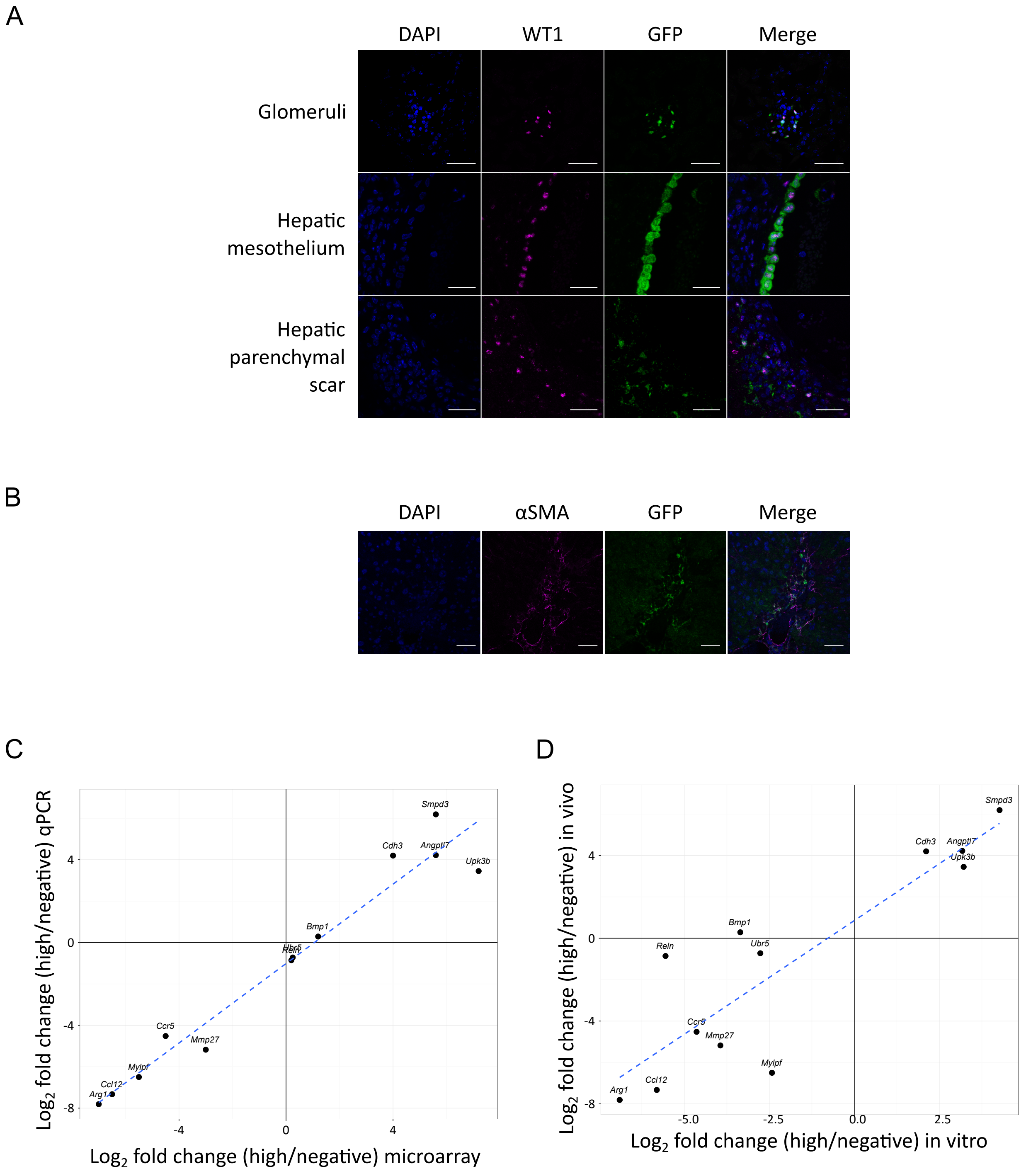

Supplementary Figure 4. GFP in WT1$^{GFP/+}$ animals colocalizes with WT1, and gene expression by aHSCs from *in vitro* and *in vivo* activation is reliably determined by microarray analysis. (A) WT1 colocalized with GFP expression in WT1$^{GFP/+}$ animals in glomerular podocytes (scale bars 50 µm), hepatic mesothelium (scale bars 25 µm), and hepatic scars (scale bars 30 µm). (B) GFP positive cells in fibrotic livers of WT1$^{GFP/+}$ animals injured by injection of CCl_4 were also αSMA positive. Scale bars 200 µm. (C) Strong positive correlation between differential expression of 12 genes in a comparison between WT1-high and WT1-negative aHSCs determined by microarray and qPCR (Spearman's rank correlation r_s=0.945, p=2.443x10^{-6}), n=3). (D) Strong positive correlation between differential expression of 12 genes in a comparison between WT1-negative and WT1-high aHSCs generated by in vitro activation or in vivo chronic injury (Spearman's rank correlation r_s=0.839, p=0.001192, n=3).

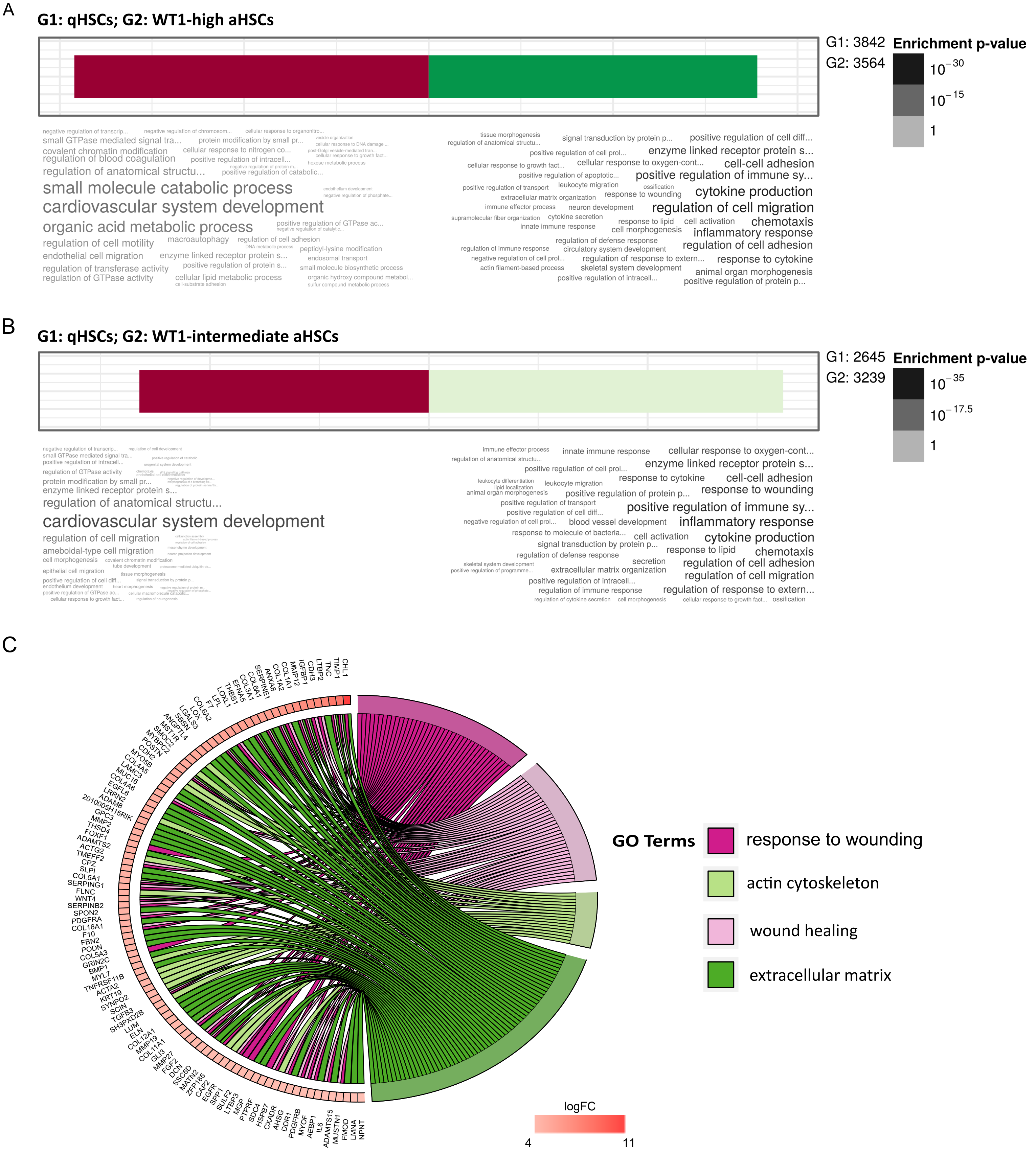

Supplementary Figure 5. Scar-orchestrating cells ultimately derived from the embryonic mesothelium represent a discrete lineage showing classical transcriptional responses during activation after injury. The transcriptome, determined by RNAseq, of aHSCs isolated from PDGFRβCre;WT1$^{GFP/fl}$ animals (n=6) with liver fibrosis induced by iterative injury with CCl_4 was compared with that of quiescent lineage-label positive HSCs from WT1$^{CreERT2/+}$;Ai14 animals (n=3) induced at E10.5. Gene ontology terms mapped to differentially expressed genes for WT1-high (A) and WT1-intermediate (B) aHSCs were determined and demonstrate engagement of cellular processes associated with the HSC activation paradigm. (C) Specific mapping of differentially-expressed genes in WT1-intermediate aHSCs to GO terms for indicative scarring responses are visualized by chord diagrams.

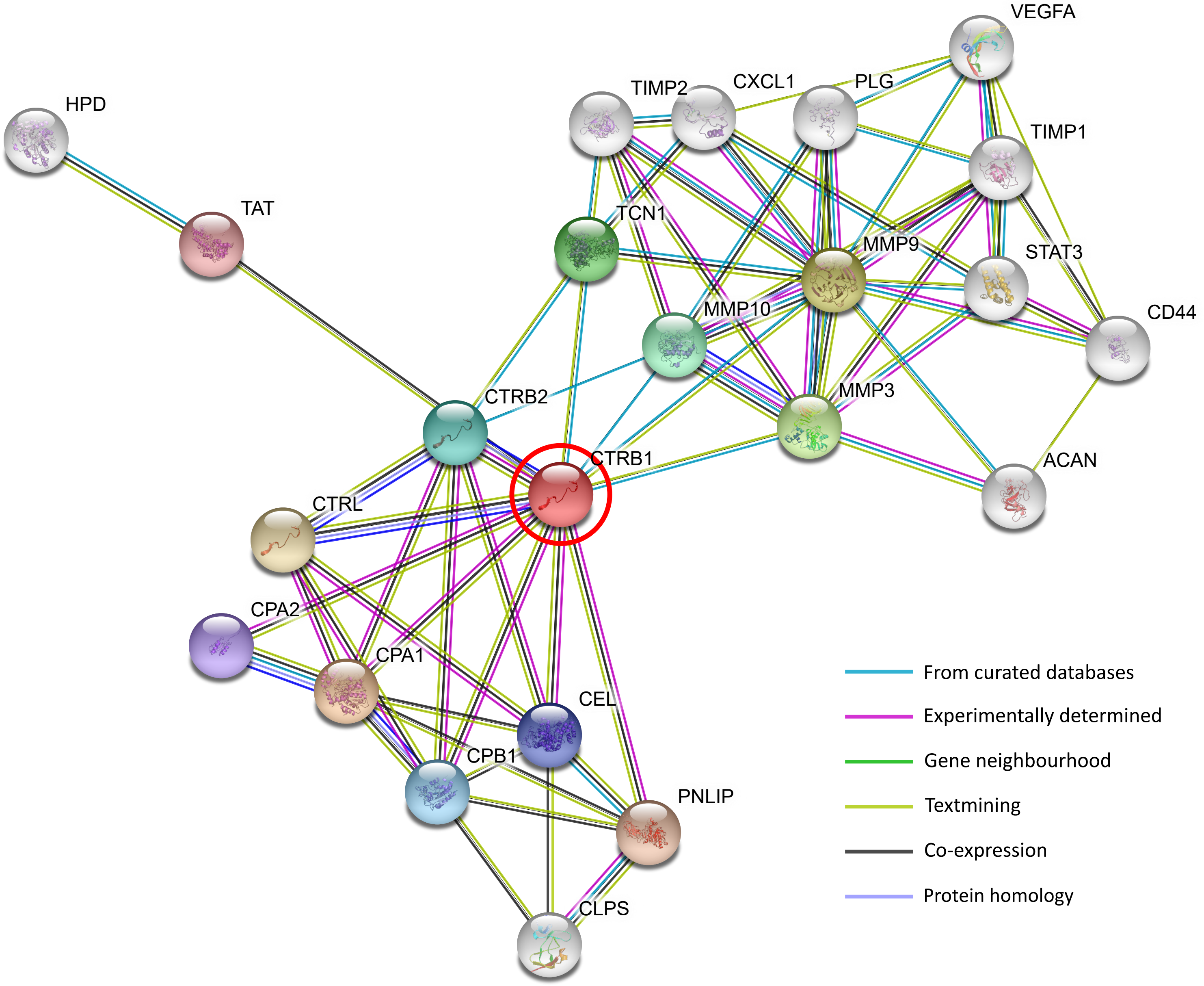

Supplementary Figure 6. Interacting proteins for CTRB1. High confidence interacting proteins for CTRB1 from the STRING database v11 web interface showing dominant interactions with pepidases and matrix metalloproteinases. Settings - network edge meaning (evidence), active interaction sources (all), minimum interaction score (high confidence 0.700), 1st shell (maximum 20 interactors), 2nd shell (maximum 10 interactors). Network diagram retrieved under CC-BY 4.0, https://version-11-0.string-db.org/cgi/network.pl?networkId=pih1Mdxrh0St.

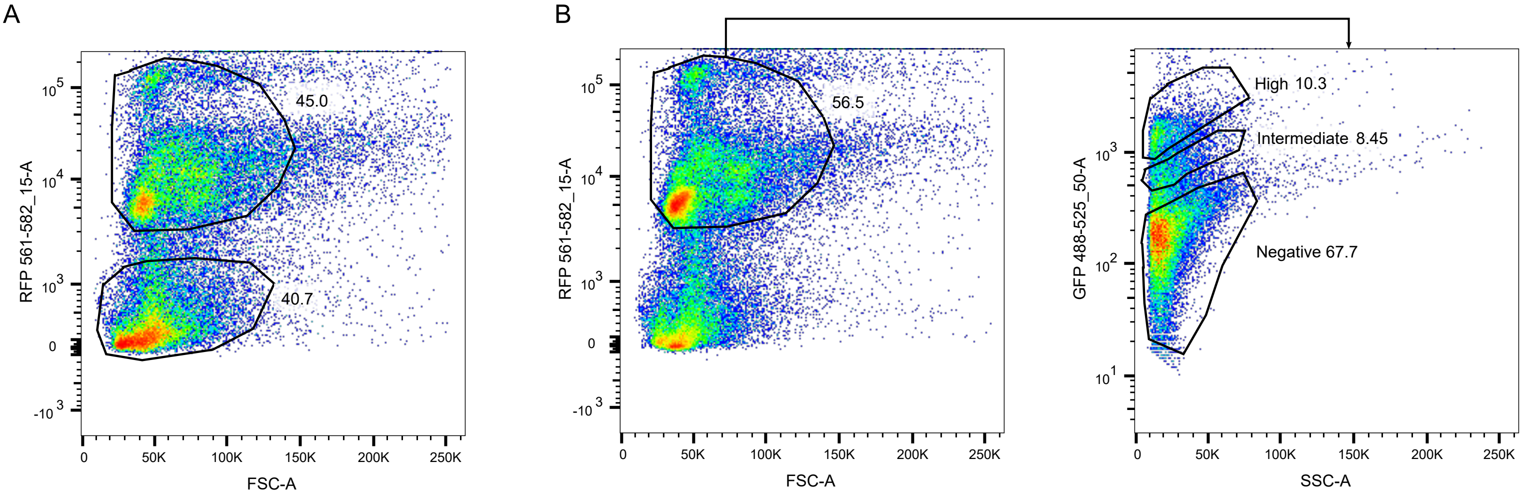

Supplementary Figure 7. Flow cytometry gating strategies of non GFP-only cell populations. (A) Sorting of Ai14 (RFP) Cre-reporter positive and negative cells. (B) Sorting of WT1(GFP)-defined populations of Ai14 (RFP) Cre-reporter positive cells.

Gene symbol	Log_2 fold change	FDR-adjusted p value	Lineage-label status differentially overexpressing population
**Col6a4**	-5.532	0.008	Negative
**Lamb3**	-4.263	0.017	Negative
**Lama2**	-2.591	<0.001	Negative
**Col8a2**	-2.489	0.015	Negative
**Lamb1**	-2.182	0.002	Negative
**Col17a1**	1.850	0.003	Positive
**Col11a2**	-1.838	0.007	Negative
**Col5a3**	-1.825	<0.001	Negative
**Col15a1**	-1.765	0.012	Negative
**Lama4**	1.646	<0.001	Positive
**Col6a2**	-1.622	<0.001	Negative
**Col4a1**	1.604	<0.001	Positive
**Col1a2**	-1.603	<0.001	Negative
**Col4a2**	1.548	0.002	Positive
**Col6a1**	-1.512	<0.001	Negative
**Col1a1**	-1.471	<0.001	Negative
**Col4a4**	1.454	<0.001	Positive
**Lama3**	1.444	<0.001	Positive
**Col13a1**	1.404	<0.001	Positive
**Col6a6**	-1.389	<0.001	Negative
**Col12a1**	-1.363	0.040	Negative
**Lamb2**	1.348	0.003	Positive
**Lama5**	1.341	0.032	Positive

**Col8a1**	1.118	0.020	Positive
**Col27a1**	1.095	0.006	Positive
**Col5a1**	-0.987	0.019	Negative
**Col14a1**	-0.960	0.008	Negative
**Col5a2**	0.797	0.016	Positive
**Lamc1**	0.677	0.032	Positive

Supplementary Table 1 - Lineage-labelled and unlabelled qHSCs differentially expressed complementary collagen and laminin species.

Gene symbol	Log2 fold change	Adjusted p value	*WT1* status of differentially overexpressing population
**Ccl11**	-4.3757	1.15E-07	Intermediate
**Lyz1**	-4.1133	1.01E-05	Intermediate
**Arg1**	-3.8641	7.5E-07	Intermediate
**Inmt**	-3.6277	1.15E-07	Intermediate
**Pf4**	-3.5117	0.000258	Intermediate
**Pcolce2**	-3.4458	2.5E-05	Intermediate
**Dpep1**	-3.4143	7.73E-07	Intermediate
**Gdf10**	-3.3972	2.85E-05	Intermediate
**Pi16**	-3.2733	2.5E-05	Intermediate
**Gsn**	-3.2345	1.82E-05	Intermediate
**Pla1a**	-2.9843	3.87E-06	Intermediate
**Ptx3**	-2.8965	5.71E-06	Intermediate
**Osr2**	-2.87	9.86E-06	Intermediate
**C1qa**	-2.8323	1.29E-07	Intermediate
**Mmp12**	-2.6339	0.004149	Intermediate
**C1qc**	-2.6202	7.69E-07	Intermediate
**C1qb**	-2.5514	2.59E-06	Intermediate
**Rarres1**	2.5402	4.29E-05	High
**Gpnmb**	-2.5071	6.36E-05	Intermediate
**Ms4a7**	-2.4677	0.000483	Intermediate

Supplementary Table 2 - Top 20 differentially expression genes in comparisons between *WT1*-high and *WT1*-intermediate subpopulations of aHSCs in vivo.

Gene symbol	Log2 fold change	Adjusted p value	*WT1* status of differentially overexpressing population
**Dmkn**	8.794	1.3269E-12	High
**Prg4**	8.307	8.3631E-13	High
**Arg1**	-8.03	1.0685E-10	Negative
**Rarres2**	7.86	8.3631E-13	High
**Krt14**	7.819	3.4678E-11	High
**Fcrls**	-7.521	5.3118E-11	Negative
**Upk1b**	7.434	6.9585E-11	High
**Upk3b**	7.306	9.8281E-11	High
**Igfbp5**	7.226	8.8402E-11	High
**Msln**	7.164	1.1249E-11	High
**Myl1**	-7.109	3.4678E-11	Negative
**Gpm6a**	6.953	8.1656E-12	High
**Has1**	6.806	2.1566E-09	High
**Cfb**	6.789	3.842E-11	High
**H2-Aa**	6.778	2.7053E-11	High
**Ccl12**	-6.758	9.2492E-08	Negative
**Gpnmb**	-6.721	2.1615E-08	Negative
**Acta1**	-6.512	2.1882E-10	Negative
**Cd74**	6.355	5.3742E-10	High
**Tnnc1**	-6.315	2.6887E-10	Negative

Supplementary Table 3 - Top 20 differentially expression genes in comparisons between *WT1* high and *WT1* negative subpopulations of aHSCs.

Gene symbol	Log2 fold change	Adjusted p value	*WT1* status of differentially overexpressing population
**Prg4**	7.9748	1.08E-12	Intermediate
**Rarres2**	7.915	1.08E-12	Intermediate
**Dmkn**	7.8704	5.36E-12	Intermediate
**Krt14**	7.2921	5.9E-11	Intermediate
**Igfbp5**	7.0261	1.4E-10	Intermediate
**H2-Aa**	6.9981	2.42E-11	Intermediate
**Upk1b**	6.9706	1.56E-10	Intermediate
**Dpt**	6.9249	2.52E-10	Intermediate
**Myl1**	-6.9241	5.78E-11	Negative
**Inmt**	6.9164	5.9E-11	Intermediate
**Cfb**	6.87	5.78E-11	Intermediate
**Gpm6a**	6.8095	1.06E-11	Intermediate
**Has1**	6.6571	3.25E-09	Intermediate
**Upk3b**	6.6196	2.99E-10	Intermediate
**Msln**	6.5455	3E-11	Intermediate
**Acta1**	-6.4914	2.65E-10	Negative
**Fcrls**	-6.4242	2.53E-10	Negative
**Tnnc1**	-6.2547	3.49E-10	Negative
**Slpi**	6.2485	1.34E-08	Intermediate
**H2-Eb1**	6.2281	5.78E-11	Intermediate

Supplementary Table 4 - Top 20 differentially expression genes in comparisons between *WT1* intermediate and *WT1* negative subpopulations of aHSCs.

KEGG pathway name	pG	pGFWER	*WT1* status of population showing pathway activation
**Chemokine signalling pathway**	4.6E-10	5.3E-08	Intermediate
**Systemic lupus erythematosus**	8.5E-10	9.9E-08	Intermediate
**Staphylococcus aureus infection**	1.1E-07	1.3E-05	Intermediate
**Complement and coagulation cascades**	8.4E-07	9.8E-05	Intermediate
**Cytokine-cytokine receptor interaction**	1.8E-05	0.00209	Intermediate
**Osteoclast differentiation**	1.8E-05	0.00211	Intermediate
**Natural killer cell mediated cytotoxicity**	3.2E-05	0.00377	Intermediate

Supplementary Table 5 - KEGG pathway perturbations in between *WT1* high and *WT1* intermediate subpopulations of aHSCs determined by signalling pathway impact analysis of differentially expressed genes (pG, p-value combining probabilities of observed number of differentially expressed genes and perturbation accumulation in pathway; pGFWER, the significance after Bonferroni correction of pG).

**KEGG pathway name**	**pG**	**pGFWER**	***WT1* status of population showing pathway activation**
**ECM-receptor interaction**	8.8E-07	0.00012	High
**Basal cell carcinoma**	4.5E-05	0.00587	High
**NF-kappa B signalling pathway**	0.0001	0.01313	Negative
**Osteoclast differentiation**	0.00026	0.03406	Negative

Supplementary Table 6 - KEGG pathway perturbations in between *WT1* high and *WT1* negative subpopulations of aHSCs determined by signalling pathway impact analysis of differentially expressed genes (pG, p-value combining probabilities of observed number of differentially expressed genes and perturbation accumulation in pathway; pGFWER, the significance after Bonferroni correction of pG).

KEGG pathway name	pG	pGFWER	*WT1* status of population showing pathway activation
**ECM-receptor interaction**	3E-06	0.00039	Intermediate
**NF-kappa B signalling pathway**	6E-06	0.00078	Negative

Supplementary Table 7 - KEGG pathway perturbations in between *WT1* intermediate and *WT1* negative subpopulations of aHSCs determined by signalling pathway impact analysis of differentially expressed genes (pG, p-value combining probabilities of observed number of differentially expressed genes and perturbation accumulation in pathway; pGFWER, the significance after Bonferroni correction of pG).

Gene symbol	Fold change	Adjusted p value	*WT1* status of differentially overexpressing population
**Ccl11**	20.759	1.15212E-07	Intermediate
**Pf4**	11.40579	0.000257526	Intermediate
**Gsn**	9.411944	1.81525E-05	Intermediate
**Mmp12**	6.207167	0.004149459	Intermediate
**Ccl9**	4.813333	2.09848E-05	Intermediate
**Mmp3**	4.517871	2.49604E-05	Intermediate
**Ccl6**	3.771895	5.08625E-05	Intermediate
**Cxcl14**	3.383029	0.003462546	Intermediate
**Ccl24**	3.313129	0.008879136	Intermediate
**Ccr5**	2.966303	0.000301686	Intermediate
**Cx3cr1**	2.916803	0.021352545	Intermediate
**Ccl4**	2.718726	0.008118698	Intermediate
**Pdgfrb**	2.433579	0.000955426	Intermediate
**Cxcl9**	2.048755	0.042485613	Intermediate
**Cxcl2**	1.87038	0.028255619	Intermediate
**Ccl7**	1.771661	0.030386658	Intermediate
**Tgfb1**	1.712899	0.02539543	Intermediate
**Mmp2**	1.564492	0.049888163	Intermediate
**Mmp19**	1.416666	0.034822591	High
**Col1a1**	N/A	N/A	N/A
**Col1a2**	N/A	N/A	N/A
**Timp1**	N/A	N/A	N/A
**Acta2**	N/A	N/A	N/A

Supplementary Table 8 - Manually-curated list of differentially expressed fibrogenic genes in comparisons between *WT1*-high and *WT1*-intermediate subpopulations of aHSCs in vivo.

Antigen	Vendor	Product number	Clonality	Species	Dilution
**GFP**	Abcam	6556	Polyclonal	Rabbit	1:1000
**PDGFRβ**	Abcam	32570	Monoclonal (Y92)	Rabbit	1:2000
**RFP**	Rockland<br>Abcam	600-401-379<br>62341	Polyclonal<br>Polyclonal	Rabbit<br>Rabbit	1:1000<br>1:200
**αSMA**	Sigma	A2547	Monoclonal (1A4)	Mouse	1:1500
**WT1**	Genetex<br>Abcam	GTX15249<br>89901	Polyclonal<br>Monoclonal (CAN-R9(IHC)-56-2)	Rabbit<br>Rabbit	1:10000<br>1:1000
**GFAP**	Dako	Z0334	Polyclonal	Rabbit	1:1500
**Desmin**	Dako	M0760	Monoclonal (D33)	Mouse	1:100
**Pan-keratin**	Dako	Z0622	Polyclonal	Rabbit	1:500

Supplementary Table 9 - Details of antibodies used.

Gene target	Forward	Reverse	UPL probe number
**18s**	cgattggatggtttagtgagg	agttcgaccgtcttctcagc	81
**Wt1**	ggacgccctacagcagtg	catctgattccaggtcatgc	33
**Timp1**	gcaaagagctttctcaaagacc	agggatagataaacagggaaacact	76
**Acta**	ccagcaccatgaagatcaag	tggaaggtagacagcgaagc	58
**Col1a1**	agacatgttcagctttgtggac	gcagctgacttcagggatg	15
**Angptl7**	gtcagtgtggtcatgcaggt	ccgagactccatgtgcttg	78
**Arg1**	gaatctgcatgggcaacc	Gaatcctggtacatctgggaac	2
**Bmp1**	cagacaagcgtcggtgtg	gtgatggagccattgagctt	27
**Ccl12**	gtccggaagctgaagagcta	Tctccttatccagtatggtcctg	71
**Ccr5**	tcatgttagatttgtacagctctcct	gtcggaactgacccttgaaa	82
**Cdh3**	ccttggaggtggaaggaact	tctccattgtctgcatggtg	1
**Mmp27**	agctgatcttcaagctgccta	tgatgacccagaaattctcatct	45
**Mylpf**	agaggatcttcgggacacct	ccactggcttccttcatcat	46
**Smpd3**	tctacctcctcgaccagcac	tgctgctccagtttgtcatc	17
**Reln**	ttctggaggacaacctcgac	tcccatctgactgacagctatg	1
**Upk3b**	ggaggggtgagctcataggt	cagttgctggtcacttggac	2
**Ubr5**	ccagaagattgccaaagctg	ggcaaaccattcccatga	69

Supplementary Table 10 - Details of qPCR primers and probes.
