# Supplementary material for: Embryonic mesothelial-derived hepatic lineage of quiescent and heterogenous scar-orchestrating cells defined but suppressed by WT1
Source: Nat Commun. 2019 Oct 15;10:4688. doi: 10.1038/s41467-019-12701-9 (PMC6794268; doi:10.1038/s41467-019-12701-9)
Supplement: Supplementary file 3 — Description of Additional Supplementary Files [file 41467_2019_12701_MOESM3_ESM.docx]

**Description of Additional Supplementary Files**

File Name: Supplementary Data 1.

Description: Differentially expressed genes in E10.5 lineage-labelled qHSCs with reference to non-lineage labelled cells.

File Name: Supplementary Data 2.

Description: Differentially activated KEGG pathways in E10.5 lineage-labelled qHSCs with reference to non-lineage labelled cells.

File Name: Supplementary Data 3.

Description: Differentially expressed genes in WT1-high with reference to WT1-intermediate subpopulations aHSCs in vivo.

File Name: Supplementary Data 4.

Description: Differentially expressed genes in WT1-high with reference to WT1-negative subpopulations of aHSCs in vivo.

File Name: Supplementary Data 5.

Description: Differentially expressed genes in WT1-intermediate with reference to WT1-negative subpopulations of aHSCs in vivo.

File Name: Supplementary Data 6.

Description: Differentially expressed genes in WT1-high aHSCs with reference to E10.5 lineage-labelled qHSCs.

File Name: Supplementary Data 7.

Description: Differentially expressed genes in WT1-intermediate aHSCs with reference to E10.5 lineage-labelled qHSCs.

File Name: Supplementary Data 8.

Description: Differentially expressed genes in GFP-high aHSCs from chronically injured PDGFRβCre;WT1GFP/fl;Ai14 animals (WT1 deletion) with reference to GFP-high cells from injured PDGFRβCre;WT1GFP/+;Ai14 aHSCs in vivo.

File Name: Supplementary Data 9.

Description: Differentially expressed genes in GFP-intermediate aHSCs from chronically injured PDGFRβCre;WT1GFP/fl;Ai14 animals (WT1 deletion) with reference to GFP-intermediate cells from injured PDGFRβCre;WT1GFP/+;Ai14 aHSCs in vivo.
