# Supplementary material for: Embryonic mesothelial-derived hepatic lineage of quiescent and heterogenous scar-orchestrating cells defined but suppressed by WT1
Source: Nat Commun. 2019 Oct 15;10:4688. doi: 10.1038/s41467-019-12701-9 (PMC6794268; doi:10.1038/s41467-019-12701-9)
Supplement: Supplementary file 2 — Peer Review [file 41467_2019_12701_MOESM2_ESM.pdf]

Reviewers' comments:

Reviewer #1 (Remarks to the Author):

Kendall et al describe functional heterogeneity of hepatic stellate cells based on Wt1 expression. The authors find that Wt1-positive and Wt1-negative HSC show different localization in the liver, a different phenotype in culture as well as different gene expression profiles. They conclude that these cells are of different origin and thereby exert differential functions in the adult liver.

1. Many parts of the paper are descriptive. The manuscript suggest that Wt1 is a marker for HSC of different origin but does not address the issue whether Wt1 could actively contribute in regulating HSC phenotype. For example, knocking out Wt1 or overexpressing Wt1 to see if cells can be converted from one state into the other are missing.

2. The different ontogeny based on WT1-CreERT2 labeling is over interpreted. It is possible that the cells have the same ontogeny, but that some subpopulations are labeled and others are no (there is also the caveat that not all cells are labeled by WT1-CreERT2; what is the labeling efficiency in this study?), or that cells divide in a hierarchical manner where Wt1-positive cells could give rise to Wt1-negative cells at the time of labeling (meaning that part of that population would remain unlabeled).

3. Parts of the manuscript on WT1-labeled cells and subcapsular fibrosis are not novel.

4. The data on different roles of the subpopulations in liver fibrosis lacks detail and functional studies. The supplementary tables with differential expression of genes suggests also a differential capacity to activate (e.g. as seen by significant differences in Cola1a and aSMA), but this is not followed up in detail. It would be important to know whether manipulation of one or the other population can indeed affect fibrosis development in vivo.

5. The authors did not investigate whether one population could differentiate into the other. Previous studies using reporter mice (Magness et al, Hepatology 2004) have suggested a high plasticity in HSC.

Minor criticims

1. The sentence “...with mesothelial-derived qHSCs having pericytic functions and qHSCs of non-mesothelial origin having immunoregulatory functions…” is too speculative.

2. It would be important to further confirm that the Msln-expressing HSC are truly HSC and not portal fibroblasts.

Reviewer #2 (Remarks to the Author):

Although heterogeneity of HSCs has been recognized, it was unknown how heterogeneous HSCs participate in liver fibrosis. The authors found that a subset of activated HSCs expresses WT1. Separation of WT1+ and WT1- HSCs from mouse livers and gene expression analysis identified differentially expressed genes in both populations. Using WT1-Cre mice, the authors showed that mesothelium contributes to WT1+ activated HSCs in liver fibrosis.

1. In Figure 4 and 5, the authors isolated Wt1 positive and negative activated HSCs from the CCl4 model and displayed a lot of differentially expressed genes. The problem of this experiment is that the authors used in vitro activated HSCs for comparison with in vivo activated HSCs. The activation of HSCs is not identical between in vivo and in vitro and the comparison made by the authors may bias genes changed by in vitro or in vivo activated states.

2. It is unclear how to isolate HSCs by FACS in Figure 4. Did the authors purify vitamin A lipid-storing HSCs by gradient centrifugation for FACS analysis? Did the author sort HSCs by the autofluorescence of vitamin A by FACS? Since HSCs show vitamin A autofluorescence, the authors can separate GFP+ Vitamin A+ and GFP- Vitamin A+ activated HSCs for comparison. Comparison of in vivo activated HSCs with or without Wt1-GFP expression will be ideal for gene expression analysis.

3. According to the description, mesothelium expresses Wt1 and both Wt1-expressing mesothelium and HSCs could be sorted by FACS in Figure 3a. In fact, mesothelium genes (Upk1b, Msln, Gpm6a) are detected by microarray analysis in Figure 4, indicating contamination of mesothelium in FACS-purified HSC population used in this study.

Thus, the inconsistency of in vitro and in vivo activation of HSCs and contamination of mesothelial cells in GFP+ HSC population by FACS make Figure 4 and 5 difficult to compare Wt1+ and Wt1- HSCs.

4. Figure 6. Did the authors purify vitamin A lipid-storing HSCs by gradient centrifugation for FACS analysis? Did the author sort HSCs by the autofluorescence of vitamin A by FACS? It seems that both RFP+ HSCs and mesothelium are sorted as RFP+ cells and this contamination may result in differential expression of genes in Figure 6e.

5. Please upload Supplementary File 3 as exel format.

6. In Figure 2, 92% of Wt1-positive HSCs express aSMA, which is a marker for activated HSCs. However, Wt1-positive HSCs isolated form the liver rather showed less expression of aSMA, ColI, and Timp1 in Figure 3. The authors did not discuss this discrepancy. As I commented above, the expression of Wt1 may be different in vivo and in vitro activated HSCs.

7. It will be desirable to examine the role of Wt1 in maintaining quiescence of HSCs by suppression of Wt1 gene in cultured HSCs.

8. Figure 1. Please indicate central vein and portal vein in the pictures. Nuclear expression of WT1 and colocalization with aSMA are not clear. Please add magnified pictures showing nuclear localization of WT1 in HSCs expressing HSC markers. In Methods, please indicate the detail of TAA, BDL, and DDC models.

9. In many figures, data labels, legends, and axis values are too small to see even in the original files. The authors should revise Figure 2c, 3a-c, 4b, and 5a and make sure all figures are large enough to see.

10. I could not read axis labels in Figure 3a. Do X- and Y-axis mean GFP and SSC? It is not clear the detail method of FACS analysis in this manuscript.

11. Discussion, lines 312. Expression of mesotheloid genes in portal fibroblasts is controversial. Mesothelial cells are often contaminated in isolated portal fibroblast population (Iwaisako et al. Proc Natl Acad Sci U S A. 2014 Aug 12;111(32):E3297-305, Lua I et al. J Hepatol. 2016 May;64(5):1137-46).

12. Methods, lines 518. No supplementary Tables were uploaded.

Reviewer #3 (Remarks to the Author):

Functional heterogeneity of scar-orchestrating cells in adult fibrotic liver injury defined by differential developmental origin

The manuscript presents interesting data and identifies a new origin of a HSC sub-population in the liver. However, the work is predominantly observational and the functional significance of WT1+ cells in fibrosis or fibrolysis is still unclear.

Figure 1: The authors should include quantification of the co-localisation of WT1 with aSMA, PDGFRb and GFAP, the co-localisation pattern appears high with PDGFRb but is not as clear with GFAP. This should also be quantified in different liver diseases including TAA, DDC and BDL.

Figure 1: The IF on human samples should be repeated to include PDGFb and GFAP co-staining with WT1 to show that the percentage of cells positive for WT1 and all 3 HSC markers used in mouse is the same in human.

The WT1+ cells are mainly absent in regions of advanced scaring in the representative images in human disease and fewer WT1+ cells are present in models of biliary disease (although there is no co-staining or matched H+E so degree of disease and fibrosis is difficult to interpret on a DAPI only channel), which is interesting.

WT1 expression increases with culture activation in about 1/3 of HM and low WT1+ expressing cells are associated with more fibrotic phenotype. Therefore I am curious to know if the 2 fold change in fibrotic genes in low WT1+ cells compared to negative versus high WT1+ cells represent a truly separate population of scar forming cell in the liver or if this reflects simply reflects plasticity of the cells and biological noise. Does WT1 directly regulate fibrotic genes and the differentially regulated genes in the microarray? Would knock-down of WT1 or overexpression of WT1 in the WT1 low expressing cells (to generate WT1 negative or high populations) switch the cells phenotype to a less fibrotic cell?

The authors use these WT1-gfp cells to transcriptionally profile WT1 negative, low and high expressing cells. A technical concern is that there is no control for GFP expression in these cells, therefore it is difficult to assess whether varying levels of GFP expression are altering the transcriptional profile of the different cell populations. This should be addressed.

Given the human data is the spatial localisation and surrounding environment important e.g. are these cells present in areas of reduced liver stiffness? What happens to the WT1+ low and high cells phenotype if cultured on plastic versus a matrix, which is less stiff, and induces quiescence e.g. matrigel? Will the WT1+ cell retain WT1 expression on matrigel?

The authors use an elegant and tamoxifen controllable system to perform lineage tracing of WT1 positive (RFP) cells to help discern the origin of the WT1+ cells and understand more about the contributions of WT1+ cells in fibrogenesis in CCl_4 injury. It will be interesting to know how the different sub populations contribute to early wound healing and fibrolysis. The authors should compare early wound healing (acute CCl_4) injury with chronic disease and recovery as well as a biliary model e.g. DDC diet or BDL.

What is the phenotype of the sub-populations of HSC in regression of fibrosis? Are these cells still present in a recovery model and do they display features of returning to a quiescent HSC state as reported by Kisseleva et al, do they become apoptotic or return to the discrete ECM phenotype described in Fig 6e.

Minor

The facs methods is unclear and need re-writing. There is no gating strategy, the x and y labels on the flojo plot isn’t labelled, one channel is GFP but the other could be anything, also what markers has been used to determine the purity of HSC? The FACS methods suggest these are freshly isolated and sorted cells but in figure 3A legend it suggests the cells are cultured for 7 days on plastic. In its current form the methods are very confusing.

The conclusion that qHSC of non-mesothelial origin having immunoregulatory functions is purely based on their gene expression profile, the immunoregulatory genes identified are not validated at a protein level and no co-culture or CM treatments of qHSC of non-mesothelial versus mesothelial origin have been performed to validate experimentally. The discussion could be reworded to reflect this.

Images of uninjured mouse and human liver are important to include.

Response to Reviewers' comments

Response to Reviewer #1

We thank the reviewer for their interest and helpful comments on our original submission. The previous editorial staff commented that whilst the reviewers found the work interesting, the main problem was the lack of experiments investigating the functional role of WT1 in fibrogenesis. Over the intervening period we have responded to this by undertaking the series of mechanistic experiments that we had always intended to do, and now submit a heavily revised new manuscript with a number of major new findings; we have recategorised the population of cells expressing less WT1 as 'WT1-intermediate' to better reflect levels of expression compared with WT1-negative cells. Where possible, we have also responded to the original specific points of each reviewer, as documented below for Reviewer #1.

Our major new findings are summarised as follows:

1. **We have deleted WT1 in hepatic stellate cells using PDGFRβCre. Paradoxically, given WT1 is only induced in activated HSCs after injury, we show that deletion of WT1 leads to an increase in fibrosis.**
2. **From a mechanistic standpoint, RNAseq analysis shows that WT1 deletion leads to a stabilisation of ECM and modulation of Wnt-signalling, favouring matrix accumulation over breakdown. Remarkably, the levels of the most down-regulated gene, CTRB1 (encoding chymotrypsinogen B), are reduced 450-fold; chymotrypsinogen B is a central node in a network of matrix degrading and stabilising proteins.**
3. **The addition of new transcriptomic data has permitted much more detailed informatic analysis of the original transcriptomic data. This confirms profound upregulation of fibrotic and wound-healing related genes in the WT1-positive HSCs during activation from their lineage-specific, mesothelial-derived WT1-negative precursor. Furthermore, a suite of genes reported to characterise the mesothelium is induced along with WT1 during this transition, reflecting the ultimate embryonic origin of this lineage.**
4. **When WT1 is deleted during activation of quiescent HSCs in cell culture, there is a morphological shift from the rounded shape towards the classical myofibroblastic shape that characterises the most fibrogenic WT1-intermediate population.**
5. **We provide several sources of evidence that we are exclusively studying HSCs and that contamination from other sources is insignificant.**

‘1. Many parts of the paper are descriptive. The manuscript suggest that Wt1 is a marker for HSC of different origin but does not address the issue whether Wt1 could actively contribute in regulating HSC phenotype. For example, knocking out Wt1 or overexpressing Wt1 to see if cells can be converted from one state into the other are missing.’

As discussed above, we have deleted WT1. We have shown that, paradoxically, fibrosis is increased after WT1 deletion, and mechanistic data suggests that this is mediated through modulation of chymotrypsinogen B and Wnt-signalling that influences matrix stability

‘2. The different ontogeny based on WT1-CreERT2 labeling is over interpreted. It is possible that the cells have the same ontogeny, but that some subpopulations are labeled and others are no (there is also the caveat that not all cells are labeled by WT1-CreERT2; what is the labeling efficiency in this study?), or that cells divide in a hierarchical manner where Wt1-positive cells could give rise to Wt1-negative cells at the time of labeling (meaning that part of that population would remain unlabeled).’

All fate-tracing experiments with induction are clearly open to the criticism that events prior to label-induction are not traced. We induced labelling at E10.5, a time-point documented in the literature indicated to allow restriction of labelling to the mesothelium[1,2].

It is implausible that labelling efficiency of any inducible label would ever reach 100% in vivo. Almost 80% of lineage-labelled activated HSCs were WT1-immunopositive after injury, as documented.

Further, the transciptomic segregation of adult quiescent HSCs based on presence or absence of E10.5 induced lineage-label was profound. The null hypothesis that all embryonic quiescent HSCs are identical and derived from the mesothelium (and that lineage-label status is only a function of Cre-efficiency), is not supported by the persistent profound differences based on lineage-status throughout adult life and period of injury of cells. Indeed, any Cre inefficiency in label-induction

would lead to 'contamination' of cells of non-mesothelial origin by mesothelial-derived, falsely-unlabelled cells; this effect will have *reduced* the strength of observed differences of lineage-labelled and -unlabelled populations in our experiments.

In vitro induction after activation of isolated quiescent HSCs, assessed by dual IF for WT1 and lineage-label, was almost 100%.

'3. Parts of the manuscript on WT1-labeled cells and subcapsular fibrosis are not novel.'

The demonstration of contribution to limited subcapsular scars has, indeed, been described before (and the work cited in this manuscript) but the examination of scarring within the deep parenchyma that makes up the bulk of the liver, rather than the limited subcapsular region, is the focus of the manuscript. However, the pertinent question that this experiment sought to answer, given the literature, was whether this mesothelial contribution was selective to subpopulations with respect to WT1-defined heterogeneity. We confirmed that the relationship between the mesothelium and WT1-positive activated HSCs is present with adult and well as embryonic origin. This has not been documented before. It is pleasing that this also functions to reproduce a previously described feature of a very high-quality paper, given the very low rates of reproducibility in biological research.

'4. The data on different roles of the subpopulations in liver fibrosis lacks detail and functional studies. The supplementary tables with differential expression of genes suggests also a differential capacity to activate (e.g. as seen by significant differences in Cola1a and aSMA), but this is not followed up in detail. It would be important to know whether manipulation of one or the other population can indeed affect fibrosis development in vivo.'

There is no means to selectively manipulate the separate WT1 populations, either separately or combined, or the WT1-negative population. Selective deletion of WT1 from these populations, with the functional implications in fibrosis, has been undertaken. RNAseq on in vivo activate and quiescent populations has been undertaken, permitting greater granularity on transcriptional profiles during activation.

5. The authors did not investigate whether one population could differentiate into the other. Previous studies using reporter mice (Magness et al, Hepatology 2004) have suggested a high plasticity in HSC.

This is a very interesting question, and one that further work, likely requiring additional labelling tools to allow manipulation of the individual WT1-defined populations, may answer.

'Minor criticisms

1. The sentence "...with mesothelial-derived qHSCs having pericytic functions and qHSCs of non-mesothelial origin having immunoregulatory functions…" is too speculative.'

We sought to summarise the differential expression of thousands of genes that is evident between quiescent HSCs dependent upon origin in as succinct a manner as possible in this manuscript documenting the lineage from development through homeostasis to adult chronic injury. Broad themes have been drawn from this large gene set using gene ontology analysis, particularly using the 'biological process' category of annotation. Even using a visualisation tool that aims to remove redundant terms, very large numbers of differentially regulated processes remain. We have provided the raw data, the derived list of differentially expressed genes and the generated differences in annotated gene ontology terms. However, we have sought to summarise the

entirety of this dataset and analysis in this manner; only a manuscript devoted entirely to this would allow fuller explanation.

‘2. It would be important to further confirm that the Msln-expressing HSC are truly HSC and not portal fibroblasts.’

By several criteria, the Msln-positive, WT1-positive activated fibrogenic cells are hepatic stellate cells. They have been isolated by classical density centrifugation due to lipid content reflective of their specialised function; other cell types are not expected to have these properties.

Further, the distribution of cells lineage-labelled at E10.5 is diffusely throughout the parenchyma in quiescence, without expression in cells within portal tracts. Given this, by design, a pericentral injury producing pericentral scarring was used for in vivo studies; injury with CCl_4 produces no periportal injury, inflammation or scarring. After injury with CCl_4, WT1-positive cells are only present in pericentral areas. WT1+ cells after in vivo injury were isolated by density centrifugation. The combination of the regional injury model (‘microarchitecturally-restricted’) used and the isolation method (‘property-restricted’) ensures that the contribution of contaminating portal-derived cells to the material study is minimal. Categorisation of cell-type, embryonic origin or histological location based upon expression of limited numbers of ‘markers’ previously attributed to other cell types is invalid.

Response to Reviewer #2

We thank the reviewer for their interest and helpful comments on our original submission. The previous editorial staff commented that whilst the reviewers found the work interesting, the main problem was the lack of experiments investigating the functional role of WT1 in fibrogenesis. Over the intervening period we have responded to this by undertaking the series of mechanistic experiments that we had always intended to do, and now submit a heavily revised new manuscript with a number of major new findings; we have recategorised the population of cells expressing less WT1 as ‘WT1-intermediate’ to better reflect levels of expression compared with WT1-negative cells. Where possible, we have also responded to the original specific points of each reviewer, as documented below for Reviewer #2.

Our major new findings are summarised as follows:

1. **We have deleted WT1 in hepatic stellate cells using PDGFRβCre. Paradoxically, given WT1 is only induced in activated HSCs after injury, we show that deletion of WT1 leads to an increase in fibrosis.**
2. **From a mechanistic standpoint, RNAseq analysis shows that WT1 deletion leads to a stabilisation of ECM and modulation of Wnt-signalling, favouring matrix accumulation over breakdown. Remarkably, the levels of the most down-regulated gene, CTRB1 (encoding chymotrypsinogen B), are reduced 450-fold; chymotrypsinogen B is a central node in a network of matrix degrading and stabilising proteins.**
3. **The addition of new transcriptomic data has permitted much more detailed informatic analysis of the original transcriptomic data. This confirms profound upregulation of fibrotic and wound-healing related genes in the WT1-positive HSCs during activation from their lineage-specific, mesothelial-derived WT1-negative precursor. Furthermore, a suite of genes reported to characterise the mesothelium is induced along with WT1 during this transition, reflecting the ultimate embryonic origin of this lineage.**

4. **When WT1 is deleted during activation of quiescent HSCs in cell culture, there is a morphological shift from the rounded shape towards the classical myofibroblastic shape that characterises the most fibrogenic WT1-intermediate population.**
5. **We provide several sources of evidence that we are exclusively studying HSCs and that contamination from other sources is insignificant.**

'1. In Figure 4 and 5, the authors isolated Wt1 positive and negative activated HSCs from the CCl4 model and displayed a lot of differentially expressed genes. The problem of this experiment is that the authors used in vitro activated HSCs for comparison with in vivo activated HSCs. The activation of HSCs is not identical between in vivo and in vitro and the comparison made by the authors may bias genes changed by in vitro or in vivo activated states.'

This is correct, and is the rationale for using material from in vivo injury wherever this is possible. However, in vitro activation by culture on plastic is frequently used when manipulations not possible in vivo are required, as is the case for this application. In this dataset, activated but WT1-cells generated by in vivo injury from the same WT1$^{+/GFP}$ animal (the ideal control) cannot be isolated for transcriptomics; collection of all WT1-negative HSCs would necessarily include quiescent cells.

The determination of differentially-expressed genes between WT1-high and WT1-intermediate populations is not affected. One of the most striking differences between WT1-positive populations and WT1-negative activated HSCs is the expression of genes previously determined to be expressed by mesothelium (although not exclusively). This finding was replicated in RNAseq from WT1-high and -intermediate activated HSCs from an additional cohort of chronically injured animals that enabled comparison with the transcriptome of quiescent HSCs. This indicates that, for one key observation central to the thesis of a unique lineage, data using an in vitro comparator is consistent with other in vivo findings.

Finally, we validated this further by additional culture activation of qHSCs from uninjured WT1$^{+/GFP}$ animals. qPCR for the set of differentially expressed genes used to validate the microarray data was undertaken on RNA derived from paired in vitro activated WT1-negative and WT1-high cells. The differential expression of the genes was determined, and compared with the differential expression of the same set of genes from the original dataset; there was strong correlation, despite supra-added intra-animal variance, indicating that an in vitro model remains valid.

'2. It is unclear how to isolate HSCs by FACS in Figure 4. Did the authors purify vitamin A lipid-storing HSCs by gradient centrifugation for FACS analysis? Did the author sort HSCs by the autofluorescence of vitamin A by FACS? Since HSCs show vitamin A autofluorescence, the authors can separate GFP+ Vitamin A+ and GFP- Vitamin A+ activated HSCs for comparison. Comparison of in vivo activated HSCs with or without Wt1-GFP expression will be ideal for gene expression analysis.'

In our laboratory, autofluorescence as a means of identification and isolation was unreliable, possibly related to specific properties/specification of the uv laser. HSCs, from injured or uninjured animals, were always isolated by density centrifugation reflecting lipid content indicative of function and, thus, designation as 'hepatic stellate cells'. A proportion of quiescent HSCs from the buoyant, isolated HSC fraction from uninjured animals were autofluorescent but a majority were not. Hence, the absence of autofluorescence could not be relied upon to identify as cell as 'non-

HSC', meaning we could not use whole non-parenchymal cell preparations and separately isolate HSCs and non-HSCs as the non-HSC fraction would have contained considerable numbers of HSCs.

'3. According to the description, mesothelium expresses Wt1 and both Wt1-expressing mesothelium and HSCs could be sorted by FACS in Figure 3a. In fact, mesothelium genes (Upk1b, Msln, Gpm6a) are detected by microarray analysis in Figure 4, indicating contamination of mesothelium in FACS-purified HSC population used in this study.

Thus, the inconsistency of in vitro and in vivo activation of HSCs and contamination of mesothelial cells in GFP+ HSC population by FACS make Figure 4 and 5 difficult to compare Wt1+ and Wt1- HSCs.'

This consideration is similar to the portal fibroblast comment of Reviewer 1, and is largely related to the categorisation of cells based on the expression of a small number of genes.

Given the apparent 'mesothelial signature' of activated WT1-positive HSCs, one explanation is contamination of the buoyant HSC fraction by mesothelial cells. One theme of the manuscript is that this is not reflective of contamination by mesothelial cells, rather a re-engagement of a signature by HSCs after injury in adulthood that is indicative of their mesothelial 'ancestry'. Confidence in the purity of isolated fibrogenic cells, with specific exclusion of hepatic mesothelial cells, is a prerequisite to this interpretation. Numerous persuasive separate lines of evidence, from our own data and the literature, have been documented to allay these concerns. Specific details in full, including additional data, are:

- **Selective isolation of fibrogenic cells.** Density centrifugation is the field standard method for hepatic stellate cell (HSC) isolation, taking advantage of buoyancy reflective of function. Buoyancy is a consequence of lipid stores, specific to these cells in the liver. The HSC field is built upon this method of isolation, and numerous prior studies have confirmed its purity.
- **Cell number.** Hepatic stellate cells are present throughout the entire substance of the liver in large numbers; the mesothelium is a monolayer only over the surface. Reflecting this, isolation of HSCs yields 50-80 times more of cells than a published specific, targeted mesothelial cell isolation method. Theoretical contamination by non-physiologically buoyant mesothelial cells, even assuming the absurd situation where their isolation was as efficient as a targeted method, can therefore account for only 1.5-2.4% of isolated cells.
- **Consistency of isolation.** The same method of isolation is used for all cell isolations, such that the same level of contamination would be expected. Theoretical mesothelial contamination cannot be used to account for the differences between subpopulations of activated HSCs whilst being accepted as absent in studies of quiescent HSCs populations (as new data) where no 'mesothelial signature' is present.
- **Complete absence of surface cell contamination.** New data has been included. Surface cells of the liver were labelled prior to disaggregation and HSC isolation; no labelled cells were identified in the fresh isolates by FACS, and none were identified during culture activation.
- **Association fallacy**. Although mesothelial cells express *WT1* (and other mesothelial-associated genes), it is false to define any cell as 'mesothelial' based on this expression alone. For example, *WT1* expression is well documented in podocytes, adipocytes, endometrial stromal cells, and myometrial smooth muscle cells, amongst others. Expression of this transcription factor is associated with profound cellular morphological

transitions; cellular expression is reflective of these critical biological functions rather than reductive 'group identity'.

'4. Figure 6. Did the authors purify vitamin A lipid-storing HSCs by gradient centrifugation for FACS analysis? Did the author sort HSCs by the autofluorescence of vitamin A by FACS? It seems that both RFP+ HSCs and mesothelium are sorted as RFP+ cells and this contamination may result in differential expression of genes in Figure 6e.'

No 'mesothelial signature' is present in quiescent HSCs, irrespective of lineage-label status, indicating no asymmetric contamination of labelled and unlabelled qHSCs by mesothelial cells that could explain the profound differences in transcriptome. For additional considerations on the unlikely roll of mesothelial contamination, please see point 3, above.

'5. Please upload Supplementary File 3 as exel format.'

Xls, xlsx and csv files can be read by most commonly used data science software, and Excel, and can be converted from one to the other.

'6. In Figure 2, 92% of Wt1-positive HSCs express aSMA, which is a marker for activated HSCs. However, Wt1-positive HSCs isolated form the liver rather showed less expression of aSMA, ColI, and Timp1 in Figure 3. The authors did not discuss this discrepancy. As I commented above, the expression of Wt1 may be different in vivo and in vitro activated HSCs.'

All HSCs in culture are aSMA positive after 7 days (the time at which populations were segregated by FACS for further examination in culture or by transcriptomics), as stated in the text. The panel referred demonstrates that WT1-high, WT1-intermediate and WT1-negative cells are 'activated' given expression of aSMA, col1a1 and Timp1 by qPCR. Critically, expression of these 3 classical 'markers' of activation and fibrogenic capacity are the same in the morphologically rounded WT1-high cells as in the classical WT1-negative myofibroblasts, and that expression is significantly higher in the WT1-intermediate myofibroblasts. Please see new Figure 6 demonstrating differential expression of classical activation genes specific to this distinct lineage.

'7. It will be desirable to examine the role of Wt1 in maintaining quiescence of HSCs by suppression of Wt1 gene in cultured HSCs.'

There is no expression of WT1 by quiescent HSCs, so WT1 cannot maintain quiescence. WT1 is induced in cells of a lineage deriving from the embryonic mesothelium only after injury during activation. The new data demonstrated that, paradoxically, WT1 deletion favours the adoption of a myofibroblast rather than rounded shape, and allows enhanced fibrogenesis after injury in vivo. This defines a new mechanism of fine control of fibrogenesis through inhibition after activation rather than simple maintenance of quiescence.

'8. Figure 1. Please indicate central vein and portal vein in the pictures. Nuclear expression of WT1 and colocalization with aSMA are not clear. Please add magnified pictures showing nuclear localization of WT1 in HSCs expressing HSC markers. In Methods, please indicate the detail of TAA, BDL, and DDC models.'

The histological literacy of the liver fibrosis field is such that we feel that annotation of such images is distracting. The citations given give the details of these models, now included in supplementary data. We can reproduce them in a supplementary methods file but have not currently done so given the number of individual figures, tables, and files currently submitted.

‘9. In many figures, data labels, legends, and axis values are too small to see even in the original files. The authors should revise Figure 2c, 3a-c, 4b, and 5a and make sure all figures are large enough to see.’

All figures in the new manuscript have modified and submitted at 500 dpi. Assuming their availability at full resolution, viewing on screen will allow zooming to a desired size.

‘10. I could not read axis labels in Figure 3a. Do X- and Y-axis mean GFP and SSC? It is not clear the detail method of FACS analysis in this manuscript.’

See point 9 re. resolution of plots. The FACS isolation is possibly misleading because of its simplicity. After standard FSC/SSC gating to exclude dead or couplet cells, native GFP only was used. No antibodies or complex gating strategy was employed.

‘11. Discussion, lines 312. Expression of mesotheloid genes in portal fibroblasts is controversial. Mesothelial cells are often contaminated in isolated portal fibroblast population (Iwaisako et al. Proc Natl Acad Sci U S A. 2014 Aug 12;111(32):E3297-305, Lua I et al. J Hepatol. 2016 May;64(5):1137-46).’

Please see response to point 3 regarding the contamination and categorisation.

‘12. Methods, lines 518. No supplementary Tables were uploaded.’

All tables are now included.

Response to Reviewer #3

We thank the reviewer for their interest and helpful comments on our original submission. The previous editorial staff commented that whilst the reviewers found the work interesting, the main problem was the lack of experiments investigating the functional role of WT1 in fibrogenesis. Over the intervening period we have responded to this by undertaking the series of mechanistic experiments that we had always intended to do, and now submit a heavily revised new manuscript with a number of major new findings; we have recategorised the population of cells expressing less WT1 as ‘WT1-intermediate’ to better reflect levels of expression compared with WT1-negative cells. Where possible, we have also responded to the original specific points of each reviewer, as documented below for Reviewer #3.

Our major new findings are summarised as follows:

1. **We have deleted WT1 in hepatic stellate cells using PDGFRβCre. Paradoxically, given WT1 is only induced in activated HSCs after injury, we show that deletion of WT1 leads to an increase in fibrosis.**
2. **From a mechanistic standpoint, RNAseq analysis shows that WT1 deletion leads to a stabilisation of ECM and modulation of Wnt-signalling, favouring matrix accumulation over breakdown. Remarkably, the levels of the most down-regulated gene, CTRB1 (encoding chymotrypsinogen B), are reduced 450-fold; chymotrypsinogen B is a central node in a network of matrix degrading and stabilising proteins.**
3. **The addition of new transcriptomic data has permitted much more detailed informatic analysis of the original transcriptomic data. This confirms profound upregulation of fibrotic and wound-healing related genes in the WT1-positive HSCs during activation from their lineage-specific, mesothelial-derived WT1-negative precursor. Furthermore, a suite of genes reported to characterise the mesothelium is induced along with WT1 during this transition, reflecting the ultimate embryonic origin of this lineage.**

4. **When WT1 is deleted during activation of quiescent HSCs in cell culture, there is a morphological shift from the rounded shape towards the classical myofibroblastic shape that characterises the most fibrogenic WT1-intermediate population.**
5. **We provide several sources of evidence that we are exclusively studying HSCs and that contamination from other sources is insignificant.**

'The manuscript presents interesting data and identifies a new origin of a HSC sub-population in the liver. However, the work is predominantly observational and the functional significance of WT1+ cells in fibrosis or fibrolysis is still unclear.'

As summarised above, we have undertaken deletion of WT1 to demonstrate an intrinsic role in activated cells to limit fibrogenic capacity. Description of the subpopulations and lineage through development, homeostasis and injury is still valuable.

'Figure 1: The authors should include quantification of the co-localisation of WT1 with aSMA, PDGFRb and GFAP, the co-localisation pattern appears high with PDGFRb but is not as clear with GFAP. This should also be quantified in different liver diseases including TAA, DDC and BDL.'

The unmanipulated images are subject to the vagaries of antibody performance, and the rabbit monoclonal used for PDGFRb staining performed very well. This is an accepted 'marker' of HSCs but we elected to include images and data from other accepted markers, with less picture-esque performance, for completeness. WT1 immunpositivity was restricted to PDGRb immunopositive cells, so not formally counted although this could be done. For the reasons documented elsewhere, a single model was pursued longitudinally and the indicative staining from additional models only remaining for reference.

'Figure 1: The IF on human samples should be repeated to include PDGFb and GFAP co-staining with WT1 to show that the percentage of cells positive for WT1 and all 3 HSC markers used in mouse is the same in human.'

As above, the murine CCl_4 model was utilised for in depth study and mechanistic manipulation in the additional data. The indicative human staining remains included to demonstrate broad relevance. The explants stained are cirrhotic and show little disease activity. A formal new study, examining larger defined cohorts to include non-cirrhotic material, stratified by aetiology, disease activity and stage, would be required to draw formal conclusions about the quantitative comparative pathology, and this is beyond the scope of the new manuscript.

'The WT1+ cells are mainly absent in regions of advanced scaring in the representative images in human disease and fewer WT1+ cells are present in models of biliary disease (although there is no co-staining or matched H+E so degree of disease and fibrosis is difficult to interpret on a DAPI only channel), which is interesting.

WT1 expression increases with culture activation in about 1/3 of HM and low WT1+ expressing cells are associated with more fibrotic phenotype. Therefore I am curious to know if the 2 fold change in fibrotic genes in low WT1+ cells compared to negative versus high WT1+ cells represent a truly separate population of scar forming cell in the liver or if this reflects simply reflects plasticity of the cells and biological noise. Does WT1 directly regulate fibrotic genes and the differentially regulated genes in the microarray? Would knock-down of WT1 or overexpression of WT1 in the WT1 low expressing cells (to generate WT1 negative or high populations) switch the cells phenotype to a less fibrotic cell?'

Our new data demonstrates that, paradoxically, WT1 deletion favours the adoption of a myofibroblast rather than rounded shape, and allows enhanced fibrogenesis after injury in vivo. This defines a new mechanism of fine control of fibrogenesis through inhibition after activation rather than simple maintenance of quiescence.

'The authors use these WT1-gfp cells to transcriptionally profile WT1 negative, low and high expressing cells. A technical concern is that there is no control for GFP expression in these cells, therefore it is difficult to assess whether varying levels of GFP expression are altering the transcriptional profile of the different cell populations. This should be addressed.'

This is a theoretical concern that is of relevance to any fluorescent reporter of an intracellular protein. However, it is difficult or impossible to address. RFP-related fluorophores derived from *Discosoma* show markedly similar tertiary structures to *Aequorea*-derived GFP-related fluorophores, given the evolutionary relationship between the parent species. Indeed, RFP chromophore maturation requires GFP-like intermediates[3]. In our data, RFP lineage-labelled and unlabelled cells from uninjured animals did not differ, based on GO term analysis of their differentially expressed genes, in 'activation' processes. Further, there was no histological scarring in the livers of animals with diffusely distributed RFP-positive cells.

The $WT1^{+/GFP}$ reporter is a knockin, leading to the allele being functionally null; this removes any concern about the promiscuity and specificity of transgene reporters. The advantage of this tool for the study of a transcription factor such as WT1, or gene transcribing a protein with no extracellular domain, is that cells can be isolated without the need for permeabilisation and fixation. This maintains viability to allow subsequent culture and manipulation, and allows isolation of usable RNA for transcriptomics.

An appropriate control would require GFP driven at different expression levels in culture systems and in vivo models, with subsequent isolation and segregation into populations defined by WT1 status. Comparison could then be made with subpopulations from $WT1^{+/GFP}$ animals. However, without a fluorochrome driven from the WT1 locus there is no means of segregating the populations.

'Given the human data is the spatial localisation and surrounding environment important e.g. are these cells present in areas of reduced liver stiffness? What happens to the WT1+ low and high cells phenotype if cultured on plastic versus a matrix, which is less stiff, and induces quiescence e.g. matrigel? Will the WT1+ cell retain WT1 expression on matrigel?'

The behaviour of subpopulations of activated HSCs defined by WT1 status in resolution from fibrosis in vivo, potentially modelled by transfer of activated HSCs from plastic to Matrigel in vitro, should be the subject of future study. However, this manuscript is limited to the lineage from E10.5 through adult homeostasis to established chronic injury.

'The authors use an elegant and tamoxifen controllable system to perform lineage tracing of WT1 positive (RFP) cells to help discern the origin of the WT1+ cells and understand more about the contributions of WT1+ cells in fibrogenesis in CCl_4 injury. It will be interesting to know how the different sub populations contribute to early wound healing and fibrolysis. The authors should compare early wound healing (acute CCl_4) injury with chronic disease and recovery as well as a biliary model e.g. DDC diet or BDL.'

The establishment of a protocol allowing labelling in early development with viable pups to trace through adulthood was challenging to establish, requiring C-section and fostering given the

systemic effects of tamoxifen on the mothers, so we are gratified that this has been described as 'elegant'.

We limited these experiments, by design, to chronic disease since this is reflective of clinical burden and of the condition of the liver in patients at the point of presentation. Whilst the very first initiating events in damage to a completely normal liver are important and interesting, we chose to focus on modelling chronic disease rather than the (more rapidly modelled) initial events.

As for the point regarding behaviour on Matrigel, modelling through recovery would be fascinating to undertake in future work, given the important demonstration by Kisselava et al that persistence and inactivation rather than simply loss by apoptosis likely informs disease progression. Similarly, per a response to Reviewer 1, we initially chose to examine a lobular rather than biliary model of injury given the distribution of lineage-labelled qHSCs, and consistently used this model for the complete documentation of the 'life-story' of this lineage as proof of principle of alternate lineages and a paradoxical inhibitory role for WT1. It would be interesting to compare with biliary injury and the role of portal fibroblasts given either concordance or dissonance would be informative. However, a compare-and-contrast study would represent a separate manuscript.

'What is the phenotype of the sub-populations of HSC in regression of fibrosis? Are these cells still present in a recovery model and do they display features of returning to a quiescent HSC state as reported by Kisseleva et al, do they become apoptotic or return to the discrete ECM phenotype described in Fig 6e.'

See above, the exploration through recovery would be incredibly interesting but represent future work.

'Minor

The facs methods is unclear and need re-writing. There is no gating strategy, the x and y labels on the flojo plot isn't labelled, one channel is GFP but the other could be anything, also what markers has been used to determine the purity of HSC? The FACS methods suggest these are freshly isolated and sorted cells but in figure 3A legend it suggests the cells are cultured for 7 days on plastic. In its current form the methods are very confusing.'

Per Reviewer 2 point 10, the FACS isolation is possibly misleading because of its simplicity. After standard FSC/SSC gating to exclude dead or couplet cells, native GFP only was used. No antibodies or complex gating strategy was employed. The plot panel could be removed and the gating 'strategy' simply documented in the text although we chose to leave it in for indicative purposes. Purity of isolated cells has been addressed above re. contamination. Isolation based buoyancy of HSCs imbued by lipid content reflective of their unique function.

'The conclusion that qHSC of non-mesothelial origin having immunoregulatory functions is purely based on their gene expression profile, the immunoregulatory genes identified are not validated at a protein level and no co-culture or CM treatments of qHSC of non-mesothelial versus mesothelial origin have been performed to validate experimentally. The discussion could be reworded to reflect this.'

Per response to a minor point raised by Reviewer 1. This is an attempt to summarise the large number of GO 'biological process' annotation changes from the large transcriptomic dataset in a short paragraph for brevity and readability. The Results and Discussion sections pertaining to this have been rewritten to accommodate the significant increase in data whilst maintaining a sensible manuscript length.

‘Images of uninjured mouse and human liver are important to include.’

Given the additional new data from knockout studies and allied refined bioinformatics, supplementary files and figures are considerable in number so we have not included images ‘showing’ an absence of WT1 immunopositivity from uninjured animals or normal human liver at this point.

1. Asahina, K., Zhou, B., Pu, W. T. & Tsukamoto, H. Septum transversum-derived mesothelium gives rise to hepatic stellate cells and perivascular mesenchymal cells in developing mouse liver. *Hepatology* **53,** 983–995 (2011).
2. Rackley, R. R. *et al.* Expression of the Wilms’ tumor suppressor gene WT1 during mouse embryogenesis. *Cell Growth Differ.* **4,** 1023–1031 (1993).
3. Wiehler, J., von Hummel, J. & Steipe, B. Mutants of Discosoma red fluorescent protein with a GFP-like chromophore. *FEBS Lett.* **487,** 384–389 (2001).

Reviewers' comments:

Reviewer #1 (Remarks to the Author):

Kendall et al have included novel experiments, in particular studies showing a role for WT1 in liver fibrosis. However, a number of concerns remain and the entire manuscript and many of the figures are extremly difficult to understand - even for a stellate cell and fibrosis expert. This is mostly due to poor description of experiments in the figure legends, but also insufficient organization of data, as well as the lack of key controls, comparisons or experiments. For example, Fig. 1B lack a quiescent control; Fig.1C lacks the negative population (shown as legend, but not visible); Figure 1 lacks in vivo models of fibrosis (WT1 is predominantly activated in vivo - only marginally in culture activation) and it is confusing to have the CCl4 data in Fig.1A to be followed by the much less relevant in vitro data; Fig.2 lack the FACS plot analyses - so it is hard to determine what precisely the authors isolated. Figure 5D list only the mesothelial genes and status of fibrogenic genes is not clear; Fig.6 does not include proper comparisons between different groups of activated cells (see also comment below).

Major points

1. The authors describe a WT1-GFP high HSC subpopulation that expresses mesothelial genes. At the same time, they also describe a WT1CreERT2-traced HSC subpopulation which also expresses high amounts of mesothelial genes. The relationship between these two are not clear, in particular since WT1 is not active in quiescent cells, meaning that some of the WT1CreERT2-traced HSC turn on WT1 only after injury. Are they the only cells that turn on WT1?

2. The authors suggest in Figure 1 that the WT1-intermediate population is the most fibrogenic - yet deletion of WT1 later on increases fibrogenesis. This does not make much sense, and may possibly be related with the in vitro activation model in Figure 1.

3. The effect of WT1 expression status and WT1 lineage on fibrogenic functions are not sufficiently investigated in this study and do not allow conclusions: intermediate cells are more fibrogenic in Figure 1, but the differential gene expression in Fig.2 does not list fibrogenic genes. Hence it appears that this is different in vivo and that WT1 status does not affect fibrogenesis. Likewise, in Fig.6 there is also no proper comparison between the WT1-positive lineage and WT1-negative lineage to see how they differ. All comparisons are made with quiescent cells and not among the different activated cell populations. With the authors suggesting that WT1 is a major determinant of HSC subpopulations, it is surprising that these analyses are missing. Also, with the authors stating that a number of genes such as mesothelin - which were thought to distinguish HSC and portal fibroblasts

(Iwaisako et al, PNAS 2014) - are expressed by WT1-positive or labeled cells it is even more important to do an extremely careful comparison.

4. The relative contribution of the WT1 high and the WT1CreERT2-traced cells appears to lack. Figure 2 (and associated files) as well as Figure 5 lack FACS analyses that show these key facts. Hence, the biological meaning and impact of much of the presented data are difficult to evaluate.

5. The data on gene expression in Fig.5D does not make sense - the authors supposedely labeled mice throgh WT1-CreERT2. Hence cells should either be labeled or not labeled. It does not make sense to have intermediate. The figure legend and the entire figure suggest that these are data from WT1-CreERT2, but the legends (like most others) is cryptic.

Minor point

1. The previous criticism that the sentence "...with mesothelial-derived qHSCs having pericytic functions and qHSCs of nonmesothelial origin having immunoregulatory functions…" is too speculative was not addressed and these statements are still in the manuscript.

Reviewer #2 (Remarks to the Author):

The authors need to correct some figures.

Minor comments

1. Fig. 1B, please label X- and Y-axes of the FACS data. Does X-axis indicate FSC?

2. Fig. 1C, the labels of each gene (X axis) are still too small to see.

3. Fig. 1D, the right figure, the labels of each column (X axis) are too small to read.

4. Fig. 5C, the labels of each column (X axis) are too small.

5. Please abbreviate hepatic stellate cells as HSCs consistently through the text.

Response to Reviewer 1.

We thank the reviewer for recognising the novelty of the data regarding the paradoxical function of WT1 in activated hepatic stellate cells in our revised manuscript. Moreover, we greatly appreciate the reviewer's detailed analysis and constructive comments that have stimulated significant revision.

We are sorry that the reviewer found the presentation and ordering of the data within the manuscript to be problematic, clearly obscuring the overall narrative and some experimental detail. We have taken this 'data communication' deficiency seriously and have comprehensively restructured the data and reordered the manuscript to present the findings in a more intuitive manner.

Below, we have provided a detailed response to each of the reviewer's comments. Briefly, in addition to reordering the manuscript to make the structure more coherent and intuitive, we have:

- Provided all controls and FACS analysis, including replotting for clarity, with supportive additional histological controls.
- Carried out more extensive analysis of gene expression differences between WT1-high and WT1-intermediate aHSC populations, as requested. This new analysis reinforces the view that these populations are distinct, with major differences including greater fibrogenic potential of the WT1-intermdiate population.
- Added consistent figure annotations and colour-coding for subpopulation and lineage clarity.

Specific points:

Kendall et al have included novel experiments, in particular studies showing a role for WT1 in liver fibrosis. However, a number of concerns remain and the entire manuscript and many of the figures are extremly difficult to understand - even for a stellate cell and fibrosis expert. This is mostly due to poor description of experiments in the figure legends, but also insufficient organization of data...

We are pleased the new data are considered novel. The data have been restructured and the manuscript reordered to present the findings more clearly and intuitively. All figures and legends have been modified to add subpopulation and lineage details, with consistent colour representation.

..., as well as the lack of key controls, comparisons or experiments. For example, Fig. 1B lack a quiescent control

We have included a representative FACS plot of a qHSC isolation from an uninjured WT1$^{GFP/+}$ animal examined before activation by culture on plastic, Supplementary Figure 3. We have also undertaken new immunofluorescent staining on liver from vehicle-only control animals to demonstrate the absence of WT1-positive quiescent HSCs in their native histological context, included in Supplementary Figure 1.

Fig.1C lacks the negative population (shown as legend, but not visible)

The WT1-negative aHSC subpopulation was present (data are normalised to this population within each replicate) but we have clarified by revising the plot. Now Figure 4B

Figure 1 lacks in vivo models of fibrosis (WT1 is predominantly activated in vivo - only marginally in culture activation) and it is confusing to have the CCl4 data in Fig.1A to be followed by the much less relevant in vitro data;

We have now emphasised that in vitro study is essential to allow examination of some cellular properties that cannot be assessed within tissue after in vivo injury (p7, para 4). We have documented that the proportion of aHSCs generated by activation in vitro that are WT1-positive models the proportion of aHSCs that are WT1+ after in vivo injury (p6, para 3 and p7, para 5). A set of genes, induced by activation after in vivo injury or culture on plastic, has been used to validate in vitro activation as a model at the transcript level (Supplementary Figure 4). In response to the reviewer's comments, we have reordered the data so that the in vivo findings are introduced first, and in vitro modelling is introduced en bloc for the necessary examination of specific cellular features.

Fig.2 lack the FACS plot analyses - so it is hard to determine what precisely the authors isolated.

Representative plots have now been included in Figure 5.

Figure 5D list only the mesothelial genes and status of fibrogenic genes is not clear. Fig.6 does not include proper comparisons between different groups of activated cells (see also comment below).

In response, we have reordered this data to emphasise the traditional activation profile of the defined mesothelial-derived lineage before documentation of the re-engagement of lineage-related mesothelial signature. We have also undertaken new targeted analysis of transcriptomics to emphasise the upregulation of genes mapping to scarring-related GO terms in WT1-intermediate versus WT1-high aHSCs beyond the global visualisation, supporting the idea of significant differences between WT1+ populations, and that WT1-intermediate are the most fibrogenic (Figure 6B). Other comparisons between subpopulations are present within the manuscript, as documented in the response below.

Major points

1. The authors describe a WT1-GFP high HSC subpopulation that expresses mesothelial genes. At the same time, they also describe a WT1CreERT2-traced HSC subpopulation which also expresses high amounts of mesothelial genes. The relationship between these two are not clear, in particular since WT1 is not active in quiescent cells, meaning that some of the WT1CreERT2-traced HSC turn on WT1 only after injury. Are they the only cells that turn on WT1?

We have documented that 78% of deep parenchymal WT1-positive aHSCs after adult injury carry the lineage label induced at E10.5, compared with only 20% of WT1-negative aHSCs, and, therefore, derive from the mesothelial-derived qHSCs traced using the $WT1^{CreERT2}$ line (Figure 2 and p5, para 2). Given neither lineage labelling by Cre induction nor immunofluorescent staining is 100% efficient, this strongly indicates significant overlap between lineage-labelled cells and WT1+ aHSCs, supporting the idea that they form a distinct lineage.

2. The authors suggest in Figure 1 that the WT1-intermediate population is the most fibrogenic - yet deletion of WT1 later on increases fibrogenesis. This does not make much sense, and may possibly be related with the in vitro activation model in Figure 1.

We were also surprised by the finding that WT1 deletion leads to increased fibrosis, however, the data are incontrovertible. Furthermore, this inverse relationship between the level WT1 expression and fibrosis is supported by the differences between the WT1-high and WT1-intermediate cells; the most fibrogenic condition is WT1 null, then WT1-intermediate, and lastly WT1-high. All these findings are consistent with the view that WT1 paradoxically marks subpopulations of aHSCs after activation but is a tonic suppressor and regulator of fibrosis. We feel that this is a novel mechanism to finely control the fibrotic response and forms the most novel conclusion of the paper.

3. The effect of WT1 expression status and WT1 lineage on fibrogenic functions are not sufficiently investigated in this study and do not allow conclusions: intermediate cells are more fibrogenic in Figure 1, but the differential gene expression in Fig.2 does not list fibrogenic genes. Hence it appears that this is different in vivo and that WT1 status does not affect fibrogenesis. Likewise, in Fig.6 there is also no proper comparison between the WT1-positive lineage and WT1-negative lineage to see how they differ. All comparisons are made with quiescent cells and not among the different activated cell populations. With the authors suggesting that WT1 is a major determinant of HSC subpopulations, it is surprising that these analyses are missing.

Please see points above in response to the comment regarding Figure 5D above. Furthermore, we have included and extended the data making these comparisons with additional analysis, and reordered within the manuscript so that they are now presented as –

- Quiescent HSC lineage-labelled v quiescent HSC non-lineage; Figure 1, Supplementary Files 1 and 2.
- Relationship of developmental mesothelial-derived lineage to adult WT1-positive aHSCs; Figure 2.
- Comparison of activated HSC populations (WT1-high, WT1-intermediate and WT1-negative); Figure 5 and Figure 6.
- Comparison of mesothelial-derived lineage qHSCs with aHSCs across the complete lineage; Figure 7, Supplementary Figure 6, Supplementary Files 6 and 7.

The complete raw transcriptomic data are deposited, and the complete lists of differentially expressed genes across all these comparisons are provided in Supplementary Files 1-7.

Also, with the authors stating that a number of genes such as mesothelin - which were thought to distinguish HSC and portal fibroblasts (Iwaisako et al, PNAS 2014) - are expressed by WT1-positive or labeled cells it is even more important to do an extremely careful comparison.

We used a density-based isolation method and pericentral injury to biologically select for HSCs distant from portal tracts. We have now included additional staining to demonstrate the absence of WT1+ cells from portal tracts after pericentral injury in Supplementary Figure 2.

4. The relative contribution of the WT1 high and the WT1CreERT2-traced cells appears to lack.

We are not sure what is meant by this comment.

Figure 2 (and associated files) as well as Figure 5 lack FACS analyses that show these key facts. Hence, the biological meaning and impact of much of the presented data are difficult to evaluate.

Example FACS data have been replotted, and in vivo examples from injured and uninjured (quiescent) $WT1^{GFP/+}$ animals are now included; Figure 4A, Figure 5B, Supplementary Figure 3.

5. The data on gene expression in Fig.5D does not make sense - the authors supposedely labeled mice throgh WT1-CreERT2. Hence cells should either be labeled or not labeled. It does not make sense to have intermediate. The figure legend and the entire figure suggest that these are data from WT1-CreERT2, but the legends (like most others) is cryptic.

The data in Figure 5D were derived from $WT1^{GFP/+}$ cells. This has been clarified by the addition of colour coding and other designators, after reordering, as Figure 8A.

Minor point

1. The previous criticism that the sentence “...with mesothelial-derived qHSCs having pericytic functions and qHSCs of nonmesothelial origin having immunoregulatory functions…” is too speculative was not addressed and these statements are still in the manuscript.

We have, accordingly, revised the short text that attempts to succinctly describe the whole-genome transcriptional differences between mesothelial-derived and non-mesothelial derived qHSC on p15, para 3.

Response to Reviewer 2

We thank the reviewer for their careful evaluation of the manuscript to identify these minor formatting errors in the Figures.

Specific points:

The authors need to correct some figures.

All figures have been revised, and formatting or scaling errors corrected.

Minor comments

1. Fig. 1B, please label X- and Y-axes of the FACS data. Does X-axis indicate FSC?

FACS plots been have been replotted, including in vivo as well as in vitro samples, in a consistent manner, with SSC on the x-axis.

2. Fig. 1C, the labels of each gene (X axis) are still too small to see.

3. Fig. 1D, the right figure, the labels of each column (X axis) are too small to read.

4. Fig. 5C, the labels of each column (X axis) are too small.

All figures revised for font size consistency across all plot elements and other figure labels.

5. Please abbreviate hepatic stellate cells as HSCs consistently through the text.

Abbreviations have been applied consistently.

Reviewers' comments:

Reviewer #1 (Remarks to the Author):

The authors have made efforts to address previous points - however, some of the key criticisms were not addressed.

Major

1. The authors did not properly address the previous criticism about the fact that major conclusions on WT1 and activation status are based on culture activation of HSC. The authors replied "We have now emphasised that in vitro study is essential to allow examination of some cellular properties that cannot be assessed within tissue after in vivo injury". While one can certainly employ a culture activation model for some aspects, it cannot be the key experiment and needs to be amended by proper in vivo investigations (e.g. isolation and comparison of in vivo activated cells - please see also comment below). Furthermore, the authors state "We were also surprised by the finding that WT1 deletion leads to increased fibrosis, however, the data are incontrovertible." The authors should consider that results are also influenced by models and that an unphysiological in vitro models may produce unphysiological results. In addition to testing the impact on HSC activation status in vivo, the authors should also demonstrate increased liver fibrosis (e.g. by Sirius Red staining). Hence, this reviewer feels that one of the central statements of the paper is not sufficiently supported by carefully performed in vivo studies.

2. The comparison in Figure 6 has not been updated to include key activation genes - which in the field constitute Col1a1, Col1a2, Acta2 and Timp1. Some of the DGE genes presented in Fig.6B are likely to be related to activation and ECM organization, but it would be essential to include above genes that are commonly accepted as standard markers of activation. These genes can be displayed, even if not differentially expressed. Without that data, one cannot conclude that WT1-intermediated HSC are more activated than WT1-high cells.

Minor:

1. It is extremely difficult to review a manuscript with all figures being unlabeled, especially with a large number of Figures and Supplementary Figures.

Response to Reviewer’s comments

We thank the reviewer for their continued interest and contribution to our submission. We have addressed the comments, as documented below, modifying the manuscript to include the suggested data and amending the text and one figure to provide further clarification.

“Major
1. The authors did not properly address the previous criticism about the fact that major conclusions on WT1 and activation status are based on culture activation of HSC. The authors replied "We have now emphasised that in vitro study is essential to allow examination of some cellular properties that cannot be assessed within tissue after in vivo injury". While one can certainly employ a culture activation model for some aspects, it cannot be the key experiment and needs to be amended by proper in vivo investigations (e.g. isolation and comparison of in vivo activated cells - please see also comment below). “

The major conclusions of the paper with respect to WT1-defined populations of aHSCs are based on unbiased transcriptomics undertaken on activated WT1-positive cells isolated after chronic injury in vivo, not in vitro activation. Whole-genome expression microarray or RNAseq has **only** been undertaken on WT1-positive populations generated by chronic injury in vivo. Fibrospatial differences between populations were also examined exclusively in vivo.

“Furthermore, the authors state "We were also surprised by the finding that WT1 deletion leads to increased fibrosis, however, the data are incontrovertible." The authors should consider that results are also influenced by models and that an unphysiological in vitro models may produce unphysiological results.”

The major conclusions of the paper with respect to the mechanistic role of WT1 in aHSCs are based on WT1-deletion in a chronic model of fibrosis in vivo and unbiased transcriptomics undertaken on activated cells isolated after chronic injury in vivo.

“In addition to testing the impact on HSC activation status in vivo, the authors should also demonstrate increased liver fibrosis (e.g. by Sirius Red staining).”

The most sensitive method to assess fibrosis in histological sections after the chronic model of fibrosis in vivo is, as stated, quantification after picrosirius red staining. This method, using whole-slide imaging and classification in FIJI, has been applied to the chronic in vivo model of fibrosis. This quantification was included in the two previous revisions in graphical form (Figure 9C in the last and current revision). We have included representative whole lobe images of PSR-stained sections in Figure 9 to highlight this and emphasised the application of the Reviewer’s suggested approach in the ‘Methods’ section of the manuscript.

C

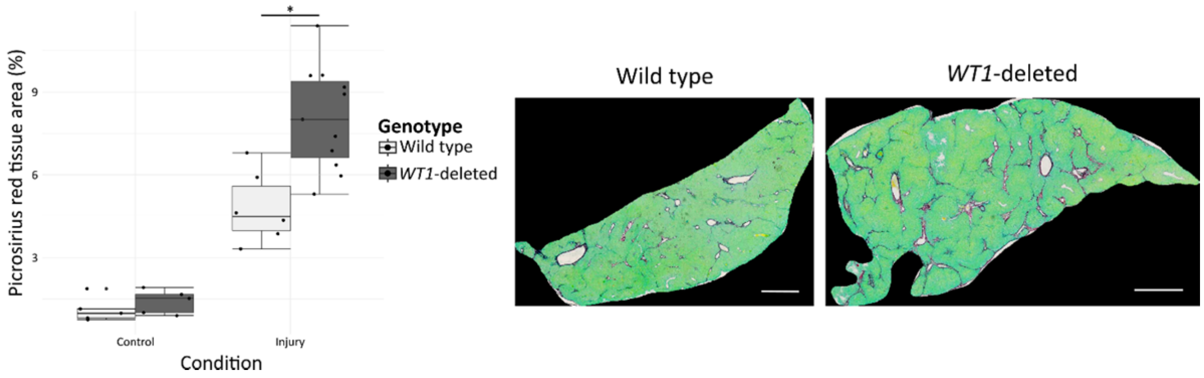

Histology and quantitative pathology

Samples of each lobe of l~~L~~ivers ~~for histological examination~~ were immediately formalin-fixed before paraffin-embedding in a single block to allow histological examination. Haematoxylin and eosin stained sections were prepared from ~~the livers of~~ each animal. ~~Picrosirius red~~PSR-stained sections were prepared ~~from injury models[42]~~[34] and whole-slide images acquired with Dotslide VS-ASW software using a motorised stage and an Olympus BX51 microscope, using an Olympus PlanApo 2X lens and Olympus XC10 camera. The PSR-positive proportion of all tissue from each whole-slide image was determined after applying the same colour threshold to all images as a script

21

within the FIJI[43] implementation of ImageJ2[44].

“Hence, this reviewer feels that one of the central statements of the paper is not sufficiently supported by carefully performed in vivo studies.”

10 main figures (Figure 1-10, inclusively) contain data. Of these, 5 (Figures 1-3, 8 and 9) are data entirely derived from in vivo work. Figures 5-7 contain transcriptomic data that, for WT1-positive populations, is also entirely derived from in vivo work. Only Figure 4 and Figure 10a contain in vitro-only data, in both cases to explicitly examine the morphological features that cannot be examined within intact tissue. Therefore, of 39 separate panels within these figures, 31 contain data derived from in vivo injury and 6 represent morphological assessment only possible in culture.

“2. The comparison in Figure 6 has not been updated to include key activation genes - which in the field constitute Col1a1, Col1a2, Acta2 and Timp1. Some of the DGE genes presented in Fig.6B are likely to be related to activation and ECM organization, but it would be essential to include above genes that are commonly accepted as standard markers of activation. These genes can be displayed, even if not differentially expressed. Without that data, one cannot conclude that WT1-intermediated HSC are more activated than WT1-high cells.”

The requested genes have been included in an additional table (Table 4) with other manually-curated differentially expressed genes that are proven to play a role in fibrogenesis. We have also discussed the implications of this in the text (Results section ‘Specific transcriptional profiles segregate with subpopulations of aHSCs defined by WT1 status and generated by fibrotic injury **in vivo**’).

This has allowed us include discussion of a range of genes that are differentially expressed by WT1-intermediate versus WT1-high aHSCs after in vivo injury and whose loss has been documented to reduce fibrogenesis in a broad range of studies. Whole-genome transcriptomics on WT1-positive populations was exclusively undertaken cells isolated from livers after chronic in vivo profibrotic injury. Given the differential expression by WT1-intermediate cells of fibrogenic-critical genes and processes, their description as having 'greater fibrogenic capacity' seems legitimate.

GO term differences between the morphologically distinct WT1-high and WT1-intermediate aHSCs were accounted for almost exclusively by overrepresentation in WT1-intermediate cells of terms relating to immune response and regulation, cell activation, response to wounding and inflammation, and chemotaxis (Figure 6A). This corresponded with activation of KEGG pathways

9

representing responses to inflammation, infection and injury (Supplementary Table 2) in WT1-intermediate cells. Explicit mappings of differentially-expressed genes to scarring-related GO terms, showing upregulation in WT1-intermediate cells in keeping with greater fibrogenic capacity, are shown in Figure 6B, including MMPs, chemokine ligands, chemokine receptors, and other genes implicated in fibrogenesis; expression of Col1a1, Col1a2, Timp1 and Acta2 are not differentially expressed. Of note, a number of the genes whose expression is upregulated in the WT1-intermediate aHSCs have been shown to be central to tissue scarring in mechanistic studies, including Pdgfrb[21], Tgfb[22], Pf4 (Cxcl4)[23], Ccl6[24], Ccl11[25], Mmp12[26], and Mmp3[27,28] (Table 4). This suggests that the transcriptionally-distinct populations of aHSCs generated by in vivo injury may influence fibrogenesis by mechanisms more complex than simple regulation of the transcription of genes encoding matrix components.

"Minor:

1. It is extremely difficult to review a manuscript with all figures being unlabeled, especially with a large number of Figures and Supplementary Figures."

The larger number of data files and figures reflect the additional work that has been included as part of the review process. Figures have always been prepared at 500 dpi and uploaded separately to maintain access at full resolution on screen. The manuscript submission system includes these as separate images at the end of the prepared pdf without applying the figure legends that are mandatory during submission of these files. The pdf, however, does create bookmarks to the image, accessible from within the document.

We agree that the representation of images in this manner is suboptimal. We have separately embedded lower-resolution images within the native Word document adjacent to their legends, whilst leaving the high-resolution versions in place to permit more detailed interrogation.

Reviewers' comments:

Reviewer #4 (Remarks to the Author):

These studies deploy various approaches to clarify the origin(s) and phenotypes of hepatic stellate cells (HSCs) in adult livers. The work is important because this has been a matter of controversy for years and clarifying the issue has potential clinical implications for preventing/treating liver fibrosis.

The results are provocative but more evidence is necessary to support some of key assumptions and consequent conclusions. Also, the new data have not been discussed in the context of prior publications which have already demonstrated HSC heterogeneity, as well as signaling pathways that modulate HSC fate in the context of adult liver injury.

More specifically:
1 - You define WT1-derived RFP (+) cells as being HSCs based on their co-expression of PDGFRb. What is the evidence that PDGFRb is exclusively expressed in HSCs? Aren't there reports indicating that cholangiocytes also express this receptor? The latter point is important given some of your other data which suggest that a sub-population of injury-activated progeny of WT1(+) cells have a "rounded" morphology and are proliferative (suggestive of proliferative epithelial cells), as well as data in Figure 8 demonstrating that the progeny of WT1(+) cells upregulate the expression of several epithelial cytokeratins, including o-called "biliary" cytokeratins 7 and 19.

2 - The immunohistochemical staining to demonstrate co-expression of WT-1 with a-sma, GFAP and other markers is not convincing. This is a key issue in light of the apparent fate changes that you observed. Were these sections co-stained or did you simply mono-stain serial sections? Higher magnification/confocal images are needed to resolve true co-expression of markers.

3 - Your data and some of the literature you cite about WT-1's role in development/cancer would suggest that WT-1 is modulating epithelial-mesenchymal transitions in HSCs. Other groups have reported that HSCs undergo such fate changes (see Rojkind and Sicklick 2006; Michelotti et al 2013, Korders and Haussinger). How do your findings complement/extend that earlier work?

4 - Evidence that HSC populations are heterogeneous is also not novel. Rojkind demonstrated this in a 1991 Lab Investigation paper in which he characterized HSC clones. The Michelotti JCI paper in 2013 reports extensive FACS characterization of primary mouse stellate cells as well as several stellate cell lines. The latter publication demonstrated that Smoothened regulated HSC transitions in viv and in vitro. In light of this background data, it is critical to determine if Smoothened regulates WT1 or WT1 regulates Smoothened.

Additional comments shared with the Editor:
I think that the evidence that deletion of WT1 attenuates liver fibrosis is solid - this appears to have been the major concern of Reviewer 1. Seems that Reviewer, I, and the authors themselves agree that it is challenging to display the vast amount of experimental data from all the omics-type analyses. This leaves the readership heavily dependent on the authors to present a balanced, unbiased summary of their results. As I stated in my review, I am extremely concerned that the authors did not provide a sufficiently balanced perspective. Although their data indicate that hepatic stellate cells are "transitional" (a.k.a., "hybrid") cells that are capable of displaying both mesenchymal and epithelial phenotypes, the authors do not articulate this as a formal conclusion or discuss this possibility in the accompanying text despite displaying these data in one of the Figures. This omission is quite curious to me given the authors' confidence in the WT1 deletion studies which prove that WT1 is a key regulator of epithelial-mesenchymal type transitions in hepatic stellate cells (as WT1 has been shown to be in other cell types). The fact that the results demonstrate that epithelial/mesenchymal fate modulation significantly impacts progression/regression of liver fibrosis merits emphasis because the role of EMT/MET and its

regulators in mediating stellate cell plasticity/heterogeneity has been extremely controversial, with several major labs in the field writing papers that explicitly deny that the process has any role in liver fibrosis but others publishing opposing results and even identifying mechanisms that regulate the process (e.g., Hedgehog, Yap, and TGFbeta signaling). Given this background, it is essential that ALL the data be presented in a balanced fashion and discussed in the context of what is already known about WT1's role as an EMT/MET regulator in development and cancer plus the current state of controversy about EMT/MET in the liver fibrosis field.

Response to Reviewer 4's comments

We thank the Reviewer for their time spent making these detailed observations. We are delighted that our data are considered 'provocative' and our submission to be important to the field, and that this is perceived to be a beneficial disruption of the current thinking to address important long-standing controversies. We are also pleased that the potential clinical benefits that may follow from this are appreciated.

"These studies deploy various approaches to clarify the origin(s) and phenotypes of hepatic stellate cells (HSCs) in adult livers. The work is important because this has been a matter of controversy for years and clarifying the issue has potential clinical implications for preventing/treating liver fibrosis.

The results are provocative but more evidence is necessary to support some of key assumptions and consequent conclusions. Also, the new data have not been discussed in the context of prior publications which have already demonstrated HSC heterogeneity, as well as signaling pathways that modulate HSC fate in the context of adult liver injury."

As the Reviewer has recognised, balanced and nuanced discussion of data in areas of such controversy is singularly challenging. We had previously taken what is clearly an overly defensive approach and so have extensively revised the Discussion in a manner that more explicitly focuses on the provocative and controversial implications, particularly with regard to HSC plasticity.

"1 - You define WT1-derived RFP (+) cells as being HSCs based on their co-expression of PDGFRb. What is the evidence that PDGFRb is exclusively expressed in HSCs? Aren't there reports indicating that cholangiocytes also express this receptor? The latter point is important given some of your other data which suggest that a sub-population of injury-activated progeny of WT1(+) cells have a "rounded" morphology and are proliferative (suggestive of proliferative epithelial cells), as well as data in Figure 8 demonstrating that the progeny of WT1(+) cells upregulate the expression of several epithelial cytokeratins, including o-called "biliary" cytokeratins 7 and 19."

We agree with the Reviewer's caution in assigning cell type exclusivity based simply on the expression of a single protein defined by antibody binding. We have integrated assessment of morphological features and histological context in combination with isolation based on functional properties to allow unbiased transciptomics to conclude that the parenchymal cells belonging to the lineage derived from the embryonic mesothelium should be considered to be HSCs, based on current definitions. The use of staining using an antibody recognising PDGFRb (and other antibodies) has aided this approach but, in itself, has not been used to ascribe cell type.

In uninjured liver, lineage-labelled cells are distributed throughout the parenchyma in a sinusoidal pattern, and the cytoplasmic expression of the lineage label allows cell morphology to be evaluated. RFP-positive cells have small cell bodies with slender, connecting, cytoplasmic processes, morphological features classically described for quiescent hepatic stellate cells. These cytological features are incompatible with histological characterisation as epithelial cells (hepatocytes or cholangiocytes forming cohesive structures) or endothelial cells. Co-expression of the lineage label with a proteins such as PDGFRb that have been defined as expressed by quiescent HSCs supports categorisation as HSCs. Additional confocal colocalisation of the lineage label with the more traditional HSC 'marker' GFAP has been undertaken to support this and included in Figure 2.

All further characterisation was undertaken after isolation of cells using the field-standard method of isolating hepatic stellate cells based on cellular buoyancy, obviously conferred by the presence of lipid droplets that are characteristic of quiescent hepatic stellate cells. The combination of histological, cytological and functional features support the assertion that a population of quiescent hepatic stellate cells is derived from the developmental mesothelium.

Expression of cytokeratins that are classically designated as 'biliary' supports the relationship of this population of cells with the mesothelium, given widespread recognition of such cytokeratin expression by the mesothelium as part of its specialised phenotype that, as its name suggests, 'sits' between mesenchyme and epithelium.

"2 - The immunohistochemical staining to demonstrate co-expression of WT-1 with a-sma, GFAP and other markers is not convincing. This is a key issue in light of the apparent fate changes that you observed. Were these sections co-stained or did you simply mono-stain serial sections? Higher magnification/confocal images are needed to resolve true co-expression of markers."

The original images were derived by both dual/triple-stained confocal and epifluorescence methods. In addition to the new confocal staining for lineage labelled cells, we have also undertaken new co-expression analysis for GFAP and desmin, and the activation 'marker' aSMA, by confocal microscopy. Where this is was a direct replication of previous data (aSMA and GFAP colocalisation) with have used an alternative WT1 antibody commonly used by the WT1 biology field to support the original conclusions. We have also completed staining to demonstrate additional supportive colocalisation of GFP with aSMA in the $WT1^{GFP/+}$ line, also by confocal microscopy. Staining has always been evaluated as part of the integrated histomorphological approach outlined above, rather than considered in isolation. This new imaging has been incorporated into Figure 3 and Supplementary Figure 4 to support or replace (in the case of Figure 3A aSMA and GFAP) the original images.

Appropriate comparative pathology assessment is required to properly evaluate disease models in a human disease context. This study has used a model of pericentral injury that recapitulates a pattern of scarring similar to that seen in steatohepatitis. Therefore, all stained histological sections and photomicrographs acquired during the work, including those present in current and prior revisions, have been reviewed for technical quality and interpreted by a practising specialist consultant liver pathologist with 17 years' experience and working at the national liver transplant centre, with additional experience reporting immunofluorescence on native and transplant medical renal biopsies, and who participates in the national liver pathology external quality assurance scheme.

"3 - Your data and some of the literature you cite about WT-1's role in development/cancer would suggest that WT-1 is modulating epithelial-mesenchymal transitions in HSCs. Other groups have reported that HSCs undergo such fate changes (see Rojkind and Sicklick 2006; Michelotti et al 2013, Korders and Haussinger). How do your findings complement/extend that earlier work?"

As the Reviewer recognises, this is one of the most important and controversial implications of our data. Explicit designation of data as demonstrating EMT has often led readers to infer additional unsupported properties based on differing understanding of that term. We had chosen to allow readers to draw those conclusions themselves as a means of navigating the controversy.

We have now included extensive formal discussion of EMT/MET in an expanded interpretation of the data with respect to cellular transitions. We agree with the Reviewer that our data indicate significant, possibly bidirectional, HSC plasticity over the full lineage that we define, clearly supporting work in this area by the groups cited. We feel that mesothelium is a sufficiently

specialised tissue, with unusual bidirectional tendencies, that mesothelium-mesenchymal transition is an appropriate label, although this could clearly represent a specialised form of EMT.

"4 - Evidence that HSC populations are heterogeneous is also not novel. Rojkind demonstrated this in a 1991 Lab Investigation paper in which he characterized HSC clones."

We have over-stated the novelty of our demonstration of heterogeneity, at least without the lineage considerations, and have now changed the Discussion to reflect this. Specifically, we propose that our definition of heterogeneity defined by distinct ancestry traced back to development is an important new consideration. The discrete lineages have properties and histological context meriting classification as HSCs, but one interpretation is that the other differences are so profound that they could be considered separate cell types. We are aware that this may move from 'provocative' towards 'heretical'.

"The Michelotti JCI paper in 2013 reports extensive FACS characterization of primary mouse stellate cells as well as several stellate cell lines. The latter publication demonstrated that Smoothened regulated HSC transitions in viv and in vitro. In light of this background data, it is critical to determine if Smoothened regulates WT1 or WT1 regulates Smoothened."

We have now specifically included the Smoothened expression throughout the defined lineage in Figure 8. Interestingly, *Smo* follows the pattern of expression shown by the other genes in the set that have a demonstrated relationship with the mesothelium. Similarly, its expression also does not change after WT1-deletion. This has allowed specific discussion of the functional implications with respect to HSC plasticity in the Discussion

"Additional comments shared with the Editor:
I think that the evidence that deletion of WT1 attenuates liver fibrosis is solid - this appears to have been the major concern of Reviewer 1. Seems that Reviewer, I, and the authors themselves agree that it is challenging to display the vast amount of experimental data from all the omics-type analyses. This leaves the readership heavily dependent on the authors to present a balanced, unbiased summary of their results. As I stated in my review, I am extremely concerned that the authors did not provide a sufficiently balanced perspective. Although their data indicate that hepatic stellate cells are "transitional" (a.k.a., "hybrid") cells that are capable of displaying both mesenchymal and epithelial phenotypes, the authors do not articulate this as a formal conclusion or discuss this possibility in the accompanying text despite displaying these data in one of the Figures. This omission is quite curious to me given the authors' confidence in the WT1 deletion studies which prove that WT1 is a key regulator of epithelial-mesenchymal type transitions in hepatic stellate cells (as WT1 has been shown to be in other cell types). The fact that the results demonstrate that epithelial/mesenchymal fate modulation significantly impacts progression/regression of liver fibrosis merits emphasis because the role of EMT/MET and its regulators in mediating stellate cell plasticity/heterogeneity has been extremely controversial, with several major labs in the field writing papers that explicitly deny that the process has any role in liver fibrosis but others publishing opposing results and even identifying mechanisms that regulate the process (e.g., Hedgehog, Yap, and TGFbeta signaling). Given this background, it is essential that ALL the data be presented in a balanced fashion and discussed in the context of what is already known about WT1's role as an EMT/MET regulator in development and cancer plus the current state of controversy about EMT/MET in the liver fibrosis field."

As documented above, we have explicitly presented our interpretation of the data with respect to HSC plasticity and EMT/MET in the Discussion, accepting that this will be considered provocative. The importance of this is reflected in our amendment of the abstract.

REVIEWERS' COMMENTS:

Reviewer #4 (Remarks to the Author):

The latest revision improves the manuscript substantially. The data presentation and discussion are comprehensive and now reflect a more balanced presentation of background information that is highly pertinent to the authors' results.

We thank Reviewer #4 for their further review and are delighted that our revisions have ‘improve[d] the manuscript substantially’ and provided a ‘balanced presentation’ of the background.

‘Reviewer #4 (Remarks to the Author):

The latest revision improves the manuscript substantially. The data presentation and discussion are comprehensive and now reflect a more balanced presentation of background information that is highly pertinent to the authors' results. ‘
